# CAFs Homologous Biomimetic Liposome Bearing BET Inhibitor and Pirfenidone Synergistically Promoting Antitumor Efficacy in Pancreatic Ductal Adenocarcinoma

**DOI:** 10.1002/advs.202305279

**Published:** 2023-11-15

**Authors:** Yin Zhang, Ranran Yu, Cheng Zhao, Jiawei Liang, Yixuan Zhang, Haochen Su, Jing Zhao, Hao Wu, Shijin Xu, Ziying Zhang, Lei Wang, Xiaoping Zou, Yun Zhu, Shu Zhang, Ying Lv

**Affiliations:** ^1^ Department of Gastroenterology Nanjing Drum Tower Hospital The Affiliated Hospital of Nanjing University Medical School Nanjing Jiangsu Province 210008 P. R. China; ^2^ Institute of Pancreatology Nanjing University Nanjing Jiangsu Province 210008 P. R. China; ^3^ State Key Laboratory of Pharmaceutical Biotechnology School of Life Sciences Nanjing University Nanjing Jiangsu Province 210008 P. R. China; ^4^ Department of Gastroenterology Nanjing Drum Tower Hospital Clinical College of Nanjing Medical University Nanjing Jiangsu Province 210008 P. R. China; ^5^ Department of Gastroenterology Nanjing Drum Tower Hospital Clinical College of Nanjing University of Chinese Medicine Nanjing Jiangsu Province 210008 P. R. China; ^6^ Department of Pharmacy Nanjing Drum Tower Hospital Drum Tower Clinical Medical College of Nanjing Medical University Nanjing Jiangsu Province 210008 P. R. China; ^7^ Nanjing Medical Center for Clinical Pharmacy Nanjing Jiangsu Province 210008 P. R. China

**Keywords:** cancer‐associated fibroblasts, nano delivery system, pancreatic cancer, tumor microenvironment

## Abstract

BRD4 is a member of the BET protein family involved in chromatin remodeling and transcriptional regulation. Several BET inhibitors (BETi) have entered clinical trials, demonstrating potential in inducing cancer cell apoptosis and tumor regression. However, resistance to BETi is common in solid tumors. In pancreatic cancer, it is found that cancer‐associated fibroblasts (CAFs) in the tumor microenvironment reduce the BET inhibitor JQ1 sensitivity by inducing BRD4 expression. Moreover, CAFs play a crucial role in the formation of a dense stromal barrier. Therefore, targeting CAFs in the tumor microenvironment of pancreatic cancer not only enhances cancer cells sensitivity to JQ1 but also increases drug perfusion and improves oxygen supply, thus reducing glycolysis and limiting energy supply. To address this challenge, a homologous targeting mechanism utilizing activated fibroblast membrane‐coated liposomes is proposed for specific drug precise target to CAFs‐rich pancreatic cancer. Additionally, TAT peptides enable liposomes penetration, delivering PFD for targeted anti‐fibrotic therapy, reducing extracellular matrix generation and glycolysis, and enhancing JQ1 delivery and sensitivity. In conclusion, the findings indicate the tremendous potential of this CAFs‐targeting liposomal delivery system in pancreatic cancer.

## Graphical abstract

This study addresses JQ1 resistance in pancreatic cancer by targeting cancer‐associated fibroblasts (CAFs). Using activated fibroblast membrane‐coated liposomes with TAT peptides, it enhances drug delivery, reduces fibrosis, glycolysis, and increases JQ1 sensitivity, offering promise for pancreatic cancer treatment.

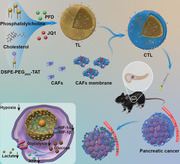

## 1 Introduction

Pancreatic cancer is a devastating malignancy characterized by a high fatality rate,^[^ ^1^ ^]^ primarily attributed to the formidable challenges in early diagnosis.^[^ ^2^ ^]^ BRD4, a member of the Bromodomain and Extraterminal (BET) protein family, is a promising target for anticancer drugs due to its involvement in organizing super‐enhancers (SEs) and regulating the expression of oncogenes.^[^ ^3^ ^]^ Inhibiting BRD4 disrupts the communication between SEs and target promoters, leading to the specific repression of oncogenes that cancer cells depend on for survival and ultimately resulting in cell death. Some BETi have not only entered clinical trials for hematologic malignancies such as AML, ALL, and CML but have also initiated clinical trials for various solid tumors, including breast cancer and colorectal cancer, demonstrating potential in inducing tumor cell apoptosis and tumor regression.^[^ ^4^ ^]^ However, resistance to BETi targeting BRD4 is common in solid tumors.^[^ ^5^ ^]^ Recent research has demonstrated that CAFs‐activated stromal signaling induces BRD4 phosphorylation in tumors, leading to increased chromatin binding and reduced binding to BETi, thus contributing to resistance. Inhibition of CAFs‐activated signaling eliminates BRD4 phosphorylation and sensitizes BETi both in vitro and in vivo.^[^ ^6^ ^]^

Furthermore, CAFs represent a major component of the pancreatic cancer tumor microenvironment.^[^ ^7^ ^]^ The fibrous extracellular matrix formed by CAFs alters the microvasculature,^[^ ^8^ ^]^ restricting the infiltration of drugs and immune cells,^[^ ^9^ ^]^ while creating a hypoxic environment necessary for the tumor's predominant energy metabolism, glycolysis.^[^ ^10^ ^]^ Targeting CAFs within the pancreatic cancer microenvironment could be an effective strategy to reduce resistance to BETi, enhance drug perfusion, improve hypoxic conditions, and suppress tumor glycolysis. Pirfenidone (PFD) is a therapeutic compound that has demonstrated efficacy in treating idiopathic pulmonary fibrosis (IPF),^[^ ^11^ ^]^ with clinically significant effects and a good safety profile in IPF patients. Recent studies have shown that PFD modulates CAFs to inhibit pancreatic tumor growth in situ and liver metastases from pancreatic tumors.^[^ ^12^ ^]^ Therefore, reducing the generation of extracellular matrix through PFD and inhibiting CAFs in the tumor tissue may potentially enhance subsequent drug perfusion and oxygen supply, and increase the sensitivity of BETi.^[^ ^13^ ^]^ Combining BETi and PFD in the treatment of pancreatic cancer may exert unique effects.

**Scheme 1:**
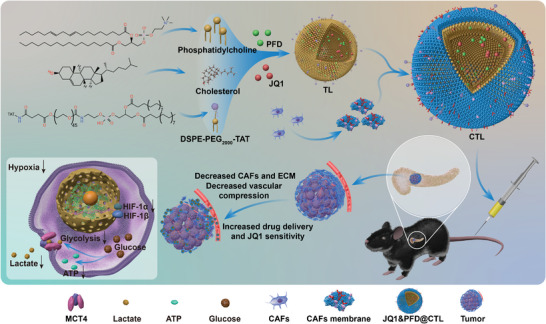
The synthesis of JQ1&PFD@CTL, along with its role in inhibiting CAFs and the formation of the stromal barrier, enhancing drug delivery, improving the hypoxic tumor environment, inhibiting tumor glycolysis, and simultaneously increasing the sensitivity of JQ1.

However, pancreatic cancer presents a significant stromal barrier, which often hampers effective drug delivery to the tumor tissue.^[^ ^14^ ^]^ Conventional administration routes lead to drug accumulation in non‐targeted tissues,^[^ ^15^ ^]^ resulting not only in limited therapeutic efficacy but also increased risks of toxic side effects.^[^ ^16^ ^]^ In this study, inspired by the significant role of CAFs in shaping the tumor microenvironment of pancreatic cancer,^[^ ^17^ ^]^ we designed a liposome‐based drug delivery system that can target tumor microenvironments of pancreatic cancer. Generally, CAFs secrete extracellular matrix to encapsulate solid tumors,^[^ ^18^ ^]^ thereby controlling the infiltration of immune cells and drugs, leading to tumor resistance.^[^ ^19^ ^]^ This provided inspiration for the design of a Trojan horse system for treating drug‐resistant tumors. We designed a novel drug delivery system that utilizes the homologous targeting mechanism to specifically target CAFs through the encapsulation of liposomes with CAFs’ cell membrane and inhibits fibroblast growth by incorporating PFD internally.^[^ ^20^ ^]^ Using cell membranes better preserves the overall biological characteristics of cell membranes, including membrane proteins, phospholipids, and various molecular components. This makes them more similar to natural biological membranes. Encapsulating liposomes with cell membranes can reduce immune reactions with the biological system, preventing them from being detected and cleared by the immune system in the bloodstream.^[^ ^21^ ^]^ Furthermore, surface modification of liposomes with TAT peptide promotes the penetration of PFD and BETi into the deeper regions of the tumor.^[^ ^22^ ^]^ As a proof of concept, animal experiments were conducted in a pancreatic cancer model encapsulated with fibrous membranes. The results indicated that liposomes prepared based on CAFs’ membrane exhibited excellent active targeting ability towards pancreatic cancer. Additionally, by targeting CAFs with PFD, the fibrous matrix in the tumor environment was disrupted, and the sensitivity of JQ1, a chemotherapy drug, was increased by modulating the impact of CAFs on BRD4, thereby enhancing the therapeutic effect of subsequent chemotherapy drugs. Transcriptome sequencing of animal tissues revealed that PFD treatment altered the tumor's hypoxic microenvironment and suppressed the tumor's glycolytic pathway, ultimately leading to disrupted energy metabolism in the tumor (**Scheme** **1**). Therefore, we believe that this Trojan horse system holds immense potential for pancreatic cancer.

## 2 Results and Discussion

### 2.1 Validation of the Mechanisms Underlying CAFs‐Mediated JQ1 Resistance in Tumors

**Figure 1:**
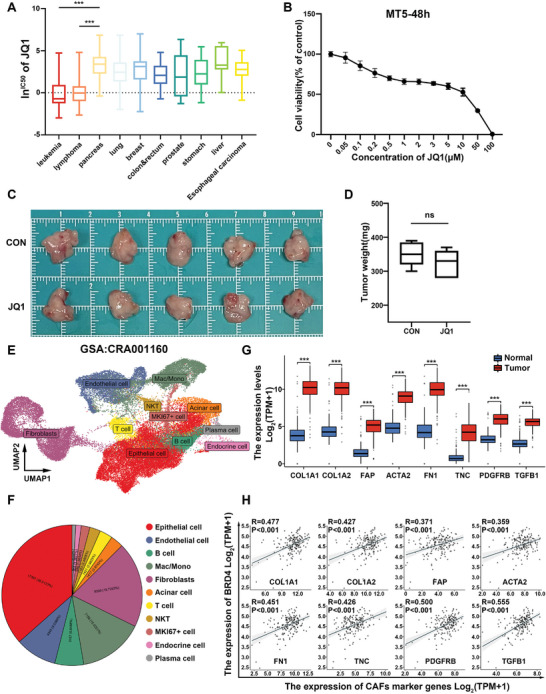
Limited efficacy of JQ1 in treating pancreatic cancer. A) After dividing the IC50 values according to tissue type in GDSC, we found that the IC50 of JQ1 was significantly higher in solid tumors than in blood system tumors, such as leukemia and lymphoma. B) MT5 cell were treated with JQ1 for 48 h, and cell viability was analyzed using a CCK‐8 kit. C) MT5 cells were transplanted into the pancreas of mice. Ten mice were divided into two groups, receiving either JQ1 (20 mg kg^−1^, i.p.) or DMSO. Treatment was administered once every 2 days for a total of seven doses. D) The weights of resected tumors from each mouse were recorded. E) UMAP shows the distribution of major cell types. The scRNA‐seq data in the study are from GSA: CRA001160. F) The percentage number of cells in each cell type. G) Integrating the TCGA‐PAAD dataset and the normal pancreatic tissue dataset from GTEx, we analyzed the expression levels of some common markers of CAFs in pancreatic cancer (*n* = 179) and normal pancreatic tissue (*n* = 171). H) Analysis of markers of CAFs in relation to BRD4 expression in TCGA‐PAAD.

First, we selected JQ1, a BET inhibitor, to assess its therapeutic efficacy in pancreatic cancer. To further validate the therapeutic potential of JQ1 in pancreatic cancer, we analyzed JQ1 data from the Genomics of Drug Sensitivity in Cancer (GDSC) database.^[^ ^23^ ^]^ Our findings indicated that solid tumors, including pancreatic cancer, breast cancer, and gastric cancer, exhibited significantly higher IC50 values for JQ1 compared to hematologic tumors, such as leukemia and lymphoma (**Figure** **1**A). Subsequently, Cell Counting Kit‐8 (CCK8) assays were employed to evaluate the impact of JQ1 treatment on murine pancreatic cancer cell line, MT5. Our results showed that after 48 h of JQ1 treatment, there was a notable effect on MT5 cells with increasing drug concentrations (Figure 1B). To determine the in vivo antitumor effects of JQ1, a mouse model with transplanted MT5 cells in the pancreas was used. Briefly, ten mice were randomly assigned to two groups: the control group and the JQ1‐treated group. However, after 2 weeks of JQ1 treatment, no significant difference in tumor growth was observed between the JQ1 treatment group and the control group (Figure 1C,D). Therefore, we speculate that the tumor microenvironment of pancreatic cancer may be a key factor leading to the reduced sensitivity of JQ1 in vivo. To explore the cellular composition of pancreatic ductal adenocarcinoma (PDAC), we utilized RNA‐seq data obtained from a study conducted by Peng et al. to generate single‐cell RNA‐seq profiles from 24 PDAC tumor samples.^[^ ^24^ ^]^ To investigate the cellular composition of the tumors, we performed principal component analysis on the differentially expressed genes across all cells, identifying 11 major clusters. Consistent with previous findings, we observed that CAFs constituted a significant proportion of the tumor tissue (Figure 1E,F). Subsequently, we examined the expression levels of specific markers for CAFs in pancreatic cancer and normal pancreatic tissue. By integrating the TCGA‐PAAD dataset with the GTEx dataset of normal pancreatic tissue, we observed significant upregulation of certain CAF‐specific marker genes in pancreatic cancer, including COL1A1, COL1A2, and ACTA2, which encode collagen I and α‐SMA (Figure 1G). This suggests that CAFs may play a crucial role in the malignant progression of pancreatic cancer. To analyze the correlation between the expression levels of genes of CAF marker genes and BRD4 in pancreatic cancer, TCGA‐PAAD dataset was processed. Our analysis revealed a positive correlation between the expression of these CAF marker genes and BRD4 (Figure 1H). Considering that BRD4 is the target of BETi, it is plausible that CAFs may contribute to the downregulation of pancreatic cancer cells' sensitivity to BETi by promoting BRD4 expression. Thus, targeting CAFs to inhibit their function represents a promising strategy for enhancing the sensitivity of JQ1 in pancreatic cancer.

### 2.2 CAFs‐Induced BRD4 Expression Drives Pancreatic Tumor Growth and Metastasis, Inhibited by PFD

**Figure 2:**
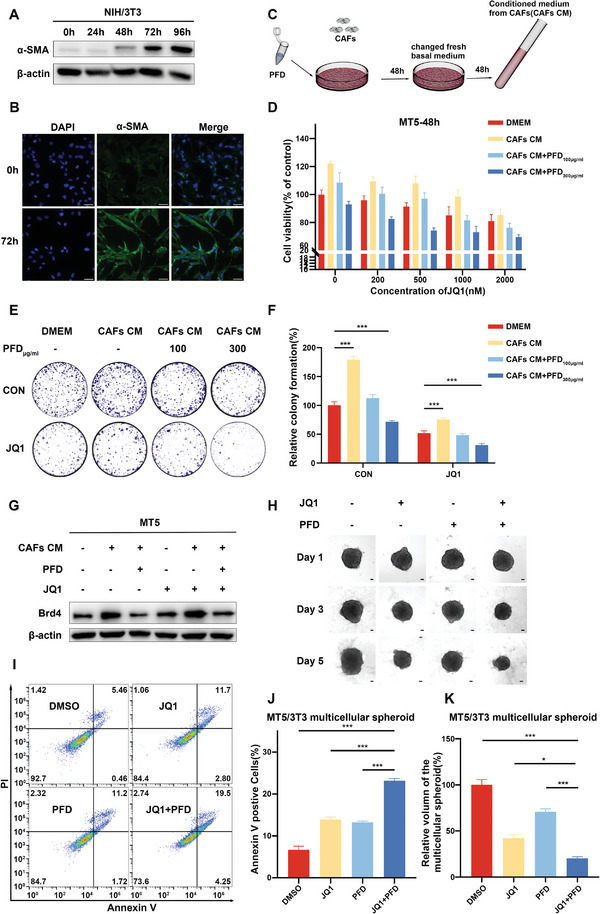
PFD inhibited CAFs‐induced proliferation of MT5 cells in vitro and increased their chemosensitivity to JQ1 treatment in pancreatic cancer. A) Western blot was used to evaluate the protein level of α‐SMA in NIH/3T3 cells at different time points after treatment with conditioned medium collected from MT5 cells. B) The expression level of α‐SMA in activated NIH/3T3 cells was evaluated by immunofluorescence staining, scale bar = 25 µm. C) Diagram of the conditioned medium obtained from CAFs (CAFs CM) or CAFs pre‐treated with PFD (CAFs CM+PFD at 100 or 300 µg mL^−1^). D) The proliferation of MT5 cells treated with JQ1 and conditioned media from CAFs or PFD‐treated CAFs was measured by CCK8 assay. E) Colony formation assay of MT5 cells treated with JQ1 and conditioned medium from CAFs or PFD‐treated CAFs. F) Quantification of the colony formation assay results. G) The expression of BRD4 protein in MT5 cells treated with JQ1 and CAFs or PFD‐treated CAFs conditioned medium was analyzed by Western blot assay. H) Representative images of MCTS treated with JQ1, PFD, and JQ1+PFD, scale bar = 80 µm. I) Antitumor efficacy of JQ1, PFD, and JQ1+PFD were measured via flow cytometry by Annexin V/PI assay. J) Quantitative analysis of the proportion of apoptosis in MCTS. K) Relative volume quantification of MCTS.

To obtain CAF cells, we cultured conditioned medium from MT5 cells with mouse embryonic fibroblasts (NIH/3T3) to activate them (CAFs).^[^ ^16b^ ^]^ Immunofluorescence showed increased α‐SMA intensity in stimulated NIH/3T3 cells compared to primary cells (**Figure** **2**B). Western blot confirmed time‐dependent α‐SMA expression increase (Figure 2A and Figure S1A, Supporting Information). These activated CAFs were successfully propagated for ten passages while maintaining high α‐SMA expression. Whether intervening with CAFs could regulate the sensitivity of pancreatic cancer cells to JQ1 was then investigated. Treatment with PFD led to a concentration‐dependent decrease in CAF proliferation, while exhibiting minimal effects on MT5 cells (Figure S2, Supporting Information). Subsequently, to assess the influence of CAFs on MT5 cells, we treated MT5 cells with conditioned medium from CAFs or PFD‐treated CAFs (Figure 2C). It is observed that conditioned medium from CAFs significantly promoted the proliferation, migration, and invasion of MT5 cells compared to normal medium. Conversely, medium from PFD‐treated CAFs reversed the proliferation, migration, and invasion induced by CAFs in MT5 cells (Figure 2D; Figures S3 and S4, Supporting Information). Furthermore, cytotoxicity and colony formation assays demonstrated that conditioned medium from PFD‐pretreated CAFs also reversed the CAF‐induced chemo‐resistance of MT5 cells to JQ1 (Figure 2D–F).

Previous studies have established a correlation between elevated levels of BRD4 and resistance to BETi.^[^ ^5^, ^25^ ^]^ In our investigation, we observed that conditioned medium from CAFs induced increased expression of BRD4 expression in MT5 cells. Both the control group and the JQ1‐treated group exhibited higher levels of BRD4 expression in the CAFs conditioned medium group compared to the normal medium group. These findings support the notion that CAFs contribute to the resistance of PDAC to BETi. Furthermore, it is observed that a downregulation of BRD4 expression in MT5 cells follows the pretreatment of CAFs with PFD (Figure 2G and Figure S1B, Supporting Information), consistent with the observed reversal of chemo‐resistance to JQ1 described earlier.

To better replicate the tumor microenvironment in vitro, a multicellular tumor spheroid (MCTS) formation assay was conducted. The previous findings demonstrated the predominant localization of activated NIH/3T3 cells outside the MCTS,^[^ ^26^ ^]^ mimicking a pathological barrier that could impede the penetration of JQ1 into the inner tumor cells. PFD, however, has the potential to enhance the penetration of JQ1 into the central region of the spheroids by reducing the stromal component, thereby improving the therapeutic efficacy of JQ1. Upon MCTS formation, DMSO, JQ1, PFD, and a combination of JQ1 and PFD were added and co‐incubated. The EVOS M7000 Imaging System was employed to capture cell sphere images (Figure 2H). After 5 days of drug treatment, the treated group exhibited a reduction in the diameter and volume of the MCTS compared to the control group. Importantly, the combination treatment group with both PFD and JQ1 showed significantly smaller tumor volumes, with a reduction of 79.79% compared to the control group, which was more effective than using PFD or JQ1 alone (Figure 2K). Subsequently, the cell spheres were dissociated into single cells for Annexin V/PI assay analysis, which revealed apoptosis in 23.75% of the cells in the co‐administered group (Figure 2I,J). In contrast, PFD exhibited only a weak anti‐tumor effect on MT5 cells. This suggests that the main mechanism of action of PFD is not direct antitumor effects but rather its ability to enhance JQ1's anti‐tumor efficacy by modulating the tumor microenvironment.

### 2.3 Charactering the Synthesis of Modified Liposomes

**Figure 3:**
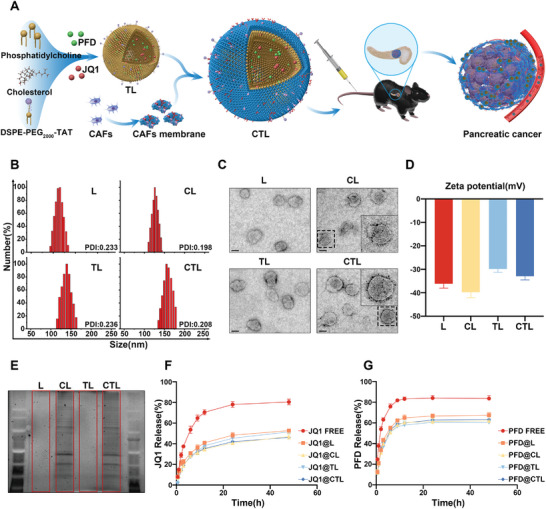
The construction and characterization of modified liposomes. A) Flow chart of CTL construction. B) Dynamic light scattering (DLS) measurements of the liposomes. C) Typical TEM images of the liposomes, scale bar = 100 nm. D) Zeta‐potential of modified liposomes. E) Protein profiles in L, CL, TL, and CTL as determined by SDS‐PAGE electrophoresis. F,G) In vitro cumulative drug release of the liposome in PBS at pH 7.4.

The pancreatic cancer microenvironment presents a notable abundance of stromal components,^[^ ^27^ ^]^ which brings a formidable hurdle for effective drug delivery. The stromal architecture serves as a physical obstruction that hampers the penetration and therapeutic efficacy of cytotoxic chemotherapies, targeted biologics, and nanomedicines.^[^ ^28^ ^]^ Traditional routes of administration often prove inadequate in delivering drugs specifically to tumor tissues, resulting in non‐target tissue accumulation. To overcome these challenges, we have leveraged the distinctive properties of CAFs within the pancreatic cancer microenvironment to develop a Trojan horse‐like liposome system. This system utilizes CAF membranes to construct liposomes, enabling homing to the tumor microenvironment and cellular membrane penetration through the surface conjugation of PEG‐linked TAT peptides (**Figure** **3**A). In essence, this Trojan horse formulation consists of liposomes bearing PEG‐linked TAT peptides on their surface (TL). These liposomes can either be encapsulated with CAF cell membranes (CL) or combined with the two components to form CAF cell membrane‐encapsulated TAT‐modified liposomes (CTL).

### 2.4 Evaluating the Targeting Efficacy of CTL on CAFs and MT5 Cells In Vitro and In Vivo

The incorporation of CAF cell membrane proteins within the liposomes imparts inherent homing properties to specifically target pancreatic cancer CAFs through a homologous targeting mechanism. Moreover, the TAT peptide facilitates cellular membrane penetration, enabling the deep penetration of PFD and BETi into the tumor's core regions. Dynamic light scattering (DLS) analysis revealed distinct average particle sizes for the modified liposomes: 115.2 ± 1.17 nm (L), 122.2 ± 1.09 nm (CL), 139.0 ± 0.83 nm (TL), and 155.5 ± 1.08 nm (CTL), while maintaining polydispersity coefficients (PDI) within an appropriate range (Figure 3B). The Zeta potentials of all groups remained within an appropriate range (Figure 3D). We observed that upon cell membrane encapsulation, CL exhibited a more negative Zeta potential compared to L, and CTL showed a more negative Zeta potential compared to TL.

This phenomenon may be attributed to the presence of negatively charged molecules, such as phospholipids and glycosylated proteins, in the cell membrane, which typically impart a negative charge to the cell membrane.^[^ ^29^ ^]^ To analyze the protein profiles of CTL, SDS‐PAGE electrophoresis was employed. The results demonstrated the retention of CAF membrane protein composition in CL and CTL, as indicated by the presence of specific protein bands, whereas no protein signals were detected in L and TL (Figure 3E). Additionally, transmission electron microscopy (TEM) images confirmed the nanoscale structure of the liposomes. A characteristic core–shell structure can be observed on the surfaces of both CL and CTL, indicating the successful encapsulation of the cell membrane on the liposome surface (Figure 3C). The average diameter of the four liposome formulations remained stable in pH 7.4 PBS over a period of 72 h (Figure S6A, Supporting Information), indicating the satisfactory stability of the prepared liposomes over time. In the presence of 10% FBS and 50% serum, all four liposome formulations remained stable within 24 h, with no significant increase in particle size (Figure S6B, Supporting Information). This suggests that the four liposome formulations exhibit good stability in the bloodstream in vivo. The encapsulation efficiencies (EE%) of PFD and JQ1 in CTL liposomes were found to be 25% and 83%, with drug loading (DL%) of 1.9% and 6.3%, respectively. To investigate the in vitro drug release kinetics, drug release experiments were conducted using a dialysis method in pH 7.4 PBS for 48 h. The released amounts of JQ1 and PFD were quantified at predetermined time intervals (Figure S5, Supporting Information). Notably, the drug‐loaded liposomes exhibited a slow release rate in pH 7.4 PBS, and no significant differences in drug release rates were observed (Figure 3F,G). In contrast, in pH 6.2 PBS, there was a significant increase in the release rate of both JQ1 and PFD, indicating rapid drug release upon reaching acidic tumor tissues (Figure S7, Supporting Information). Furthermore, cytotoxicity studies demonstrated that liposomes without encapsulated drugs did not exert detrimental effects on cell viability (Figure S8, Supporting Information), highlighting the potential of liposomes as a safe and biocompatible platform for drug delivery.

**Figure 4:**
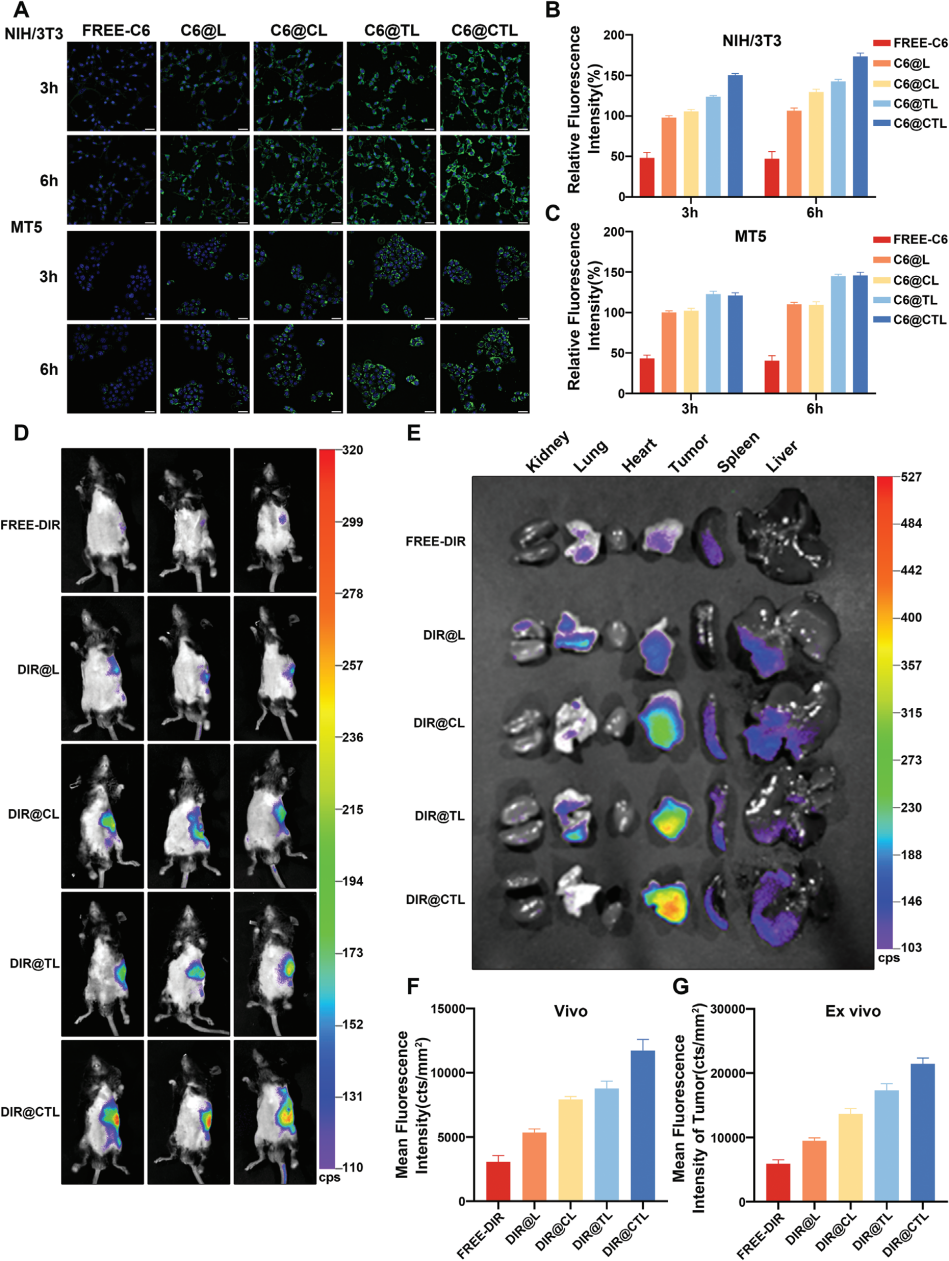
Cellular uptake ability of CTL in vitro and in vivo. A) Fluorescence images showing the uptake of FREE‐C6, C6@L, C6@CL, C6 @TL, and C6@CTL by NIH/3T3 and MT5 cells. Green fluorescence represents the encapsulated coumarin 6, blue fluorescence represents DAPI, scale bar = 50 µm. B,C) Quantification of relative fluorescence intensity. D) Fluorescence intensity of pancreatic cancer in situ lesions detected 24 hours after injection of FREE‐DIR, DIR@L, DIR@CL, DIR@TL, and DIR@CTL using the IndiGO imaging system. E) After 24 h of injection, typical ex vivo imaging of major organs in each group. F,G) Quantitative analysis of fluorescence intensity in tumors both in vivo and ex vivo for each group.

To investigate the cellular uptake and intracellular release of modified liposomes in CAFs and tumor cells, activated NIH/3T3 cells and the mouse pancreatic cancer cell line MT5 were selected for subsequent experiments. Following a 6‐h co‐incubation, the CTL group exhibited the strongest fluorescence signal in CAF cells, showing a remarkable increase of 62.84% compared to the L group and an increase of 21.42% compared to the TL group. (**Figure** **4**A,B). Compared to the L group, both the CL and TL groups displayed enhanced fluorescence signals, indicating improved cellular uptake in CAF cells mediated by the CAF cell membrane and TAT peptide. In contrast, the FREE‐C6 group showed almost no observable fluorescence. Similarly, in MT5 cells, the FREE‐C6 group exhibited nearly no detectable fluorescent signal. When comparing the CL group to the L group, the fluorescent signal remained relatively unchanged. In comparison to the L group, both the TL and CTL groups showed increased fluorescent signals, with increments of 31.85% and 32.53%, respectively. However, the difference in fluorescent signals between the TL and CTL groups was not statistically significant (Figure 4C). These results suggest that liposomes encapsulating the CAF cell membrane possess specific targeting capabilities toward CAF cells. Flow cytometry data also corroborated the findings from fluorescence microscopy (Figure S9, Supporting Information).

To analyze the in vivo behavior of the modified liposomes, MT5 cells and CAFs were co‐transplanted into the pancreas of mice to establish a pancreatic in situ graft tumor. Subsequently, liposomes loaded with a near‐infrared fluorescent probe (DIR) were intravenously injected into the mice. The distribution of liposomes was monitored at predetermined time points using the IndiGO imaging system. After 24 h post‐injection, the CTL group exhibited the highest fluorescence intensity, showing an increase of 118.79% compared to the L group. In contrast, the FREE‐DIR group showed almost no observable fluorescence. The CL and TL groups also displayed increased fluorescence signals, with increments of 47.94% and 63.72%, respectively, compared to the L group. (Figure 4D,F). Tumor tissues were then dissected, and major organs were imaged to monitor the in vivo distribution behavior of the CTL (Figure 4E,G). The fluorescence was predominantly localized in the tumor tissue. Consistent with in vivo live imaging results, the CTL group exhibited the highest fluorescence intensity, and the CL and TL groups also showed increased fluorescence signals. CTL demonstrates the strongest targeting and accumulation mechanism, primarily attributed to the encapsulation of CAFs' cell membrane, enabling CTL to more effectively accumulate in CAF‐rich pancreatic cancer tissue through homologous targeting. Upon reaching the slightly acidic tumor tissue, CTL releases its internalized TL (Figure S10, Supporting Information), and subsequently, TL, under the mediation of the TAT peptide, facilitates the lipid vesicles' easier penetration of the cell membrane, delivering the drug into the cells. The fluorescence intensity in the liver and kidney remained relatively low in all groups after 24 h post‐injection (Figure 4E and Figure S11, Supporting Information). Subsequently, we investigated the in vivo distribution of free DIR, DIR@L, and DIR@CTL at 4 and 8 h post‐injection. In vivo imaging revealed effective accumulation of DIR@L and DIR@CTL at the tumor site at 4 h post‐injection compared to free DIR, but a decrease in fluorescence intensity was observed for DIR@L at 8 hours post‐injection, while DIR@CTL continued to show significant distribution (Figure S12A, Supporting Information). In vitro imaging of major organs at 8 hours post‐injection revealed that, compared to 8 hours post‐injection, the fluorescence intensity in the liver and kidneys of the DIR@L and DIR@CTL groups decreased at 24 h post‐injection (Figure 4E; Figures S11 and S12B, Supporting Information). This suggests that at 24 h post‐injection, most of the liposomes in the liver and kidneys have been cleared. This also reflects the excellent safety profile of CTL. These findings underscore the excellent active targeting ability of TAT‐modified lipid vesicles encapsulating CAFs' cell membrane. The biological significance of these findings in pancreatic cancer targeted therapy and their potential in clinical treatment are highlighted.

### 2.5 In Vivo Evaluation of Antitumor Efficacy

**Figure 5:**
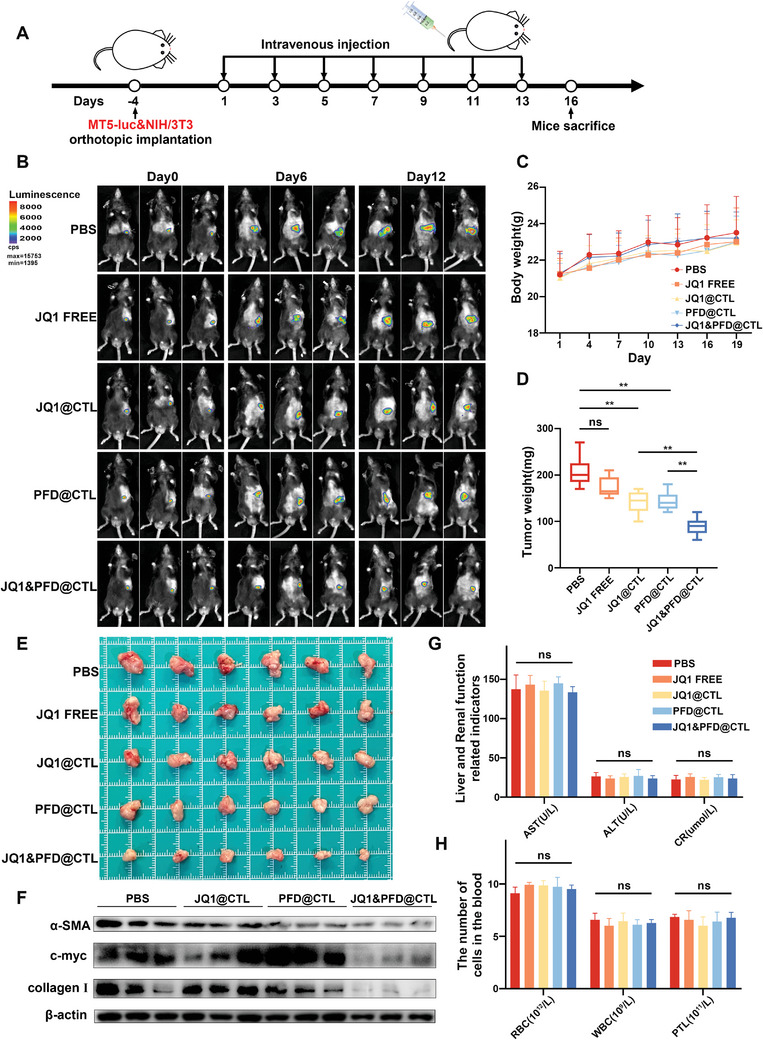
Antitumor efficacy of JQ1&PFD@CTL against pancreatic cancer in vivo. A) Schematic diagram of the mouse orthotopic pancreatic cancer transplantation model establishment and drug treatment protocol. B) Mice were divided into five groups and treated with PBS, JQ1‐FREE, JQ1@CTL, PFD@CTL, and JQ1&PFD@CTL respectively. Using luciferase in vivo imaging to dynamically monitor the size of pancreatic tumors treated with PBS, JQ1‐FREE, JQ1@CTL, PFD@CTL, and JQ1&PFD@CTL. C) The body weights of each mouse were recorded. D) The weights of resected tumors from each mouse were recorded. E) Dissected tumors after humanitarian execution. F) Western blot analysis was performed to determine the expression of α‐SMA, Collagen I, and c‐myc in each group in mouse tumor tissues. G,H) Whole blood cell analysis and evaluation of liver and renal function of mice after treatment.

To mimic the pancreatic cancer microenvironment, MT5‐luc cells and CAFs were co‐transplanted into the pancreatic tissue of mice (**Figure** **5**A). Following a 5‐day implantation period, luciferase imaging confirmed the successful formation of tumors with uniform sizes. The mice were randomly allocated to five groups and subjected to intravenous injections every 2 days for seven consecutive doses of free JQ1, JQ1@CTL, PFD@CTL, JQ1&PFD@CTL, or PBS control. Luciferase imaging was performed on days 6 and 12 to dynamically evaluate changes in tumor size (Figure 5B). After 16 days, the mice were euthanized. We observed that compared to the PBS group, the free JQ1 group did not significantly inhibit tumor growth. However, using CTL encapsulating JQ1 significantly suppressed tumor growth, indicating CTL's targeted delivery of JQ1 into the tumor, leading to enhanced perfusion, whereas only a small amount of JQ1 distributed into the tumor tissue in the free JQ1 group. The PFD@CTL group also exhibited tumor inhibition compared to the PBS group. This effect could be attributed to PFD's suppression of CAFs, thus eliminating CAFs' promoting effect on pancreatic cancer cells, rather than directly affecting cancer cells themselves. Compared to all the other groups, the JQ1&PFD@CTL group demonstrated the most potent antitumor effect, surpassing the individual use of JQ1@CTL and PFD@CTL, indicating a synergistic effect of JQ1 and PFD on pancreatic tumors. This can be attributed to two main factors. On the one hand, CTL enhanced the active targeting effect of both JQ1 and PFD. On the other hand, PFD not only reduced the generation of the extracellular matrix, thereby enhancing subsequent drug perfusion, but it also increased the sensitivity of tumor cells to JQ1, (Figure 5D,E and Figure S13, Supporting Information). Western blot analysis was employed to assess the expression levels of α‐SMA, collagen I, and c‐MYC in the tumor (Figure 5F and Figure S1C, Supporting Information). The result suggested that among the four drug intervention groups, the JQ1&PFD@CTL group exhibited the lowest expression of α‐SMA and collagen I. The expression of these two proteins in the tumor microenvironment is closely associated with the activity of CAFs and collagen deposition.^[^ ^30^ ^]^ This suggests that treatment with JQ1&PFD@CTL reduces the activity of CAFs and diminishes collagen generation. This finding is of great significance to our research, as the activity of CAFs and collagen deposition are key indicators of fibrosis in the tumor microenvironment, closely linked to tumor growth and invasion.^[^ ^31^ ^]^ BRD4 is one of the transcriptional regulatory factors of c‐MYC, an important oncogene overexpressed in various cancers.^[^ ^32^ ^]^ JQ1&PFD@CTL treatment leads to the downregulation of c‐MYC, possibly due to increased sensitivity of JQ1 to BRD4 after the suppression of CAFs' activity in the tumor microenvironment, thereby increasing JQ1's inhibitory effect on the binding of BRD4 to acetylated chromatin and its transcriptional activity,^[^ ^3a^ ^]^ ultimately reducing c‐MYC transcription. Additionally, we also assessed the expression levels of BRD4 and another CAFs marker, FAP. We observed a significant reduction in the expression levels of both BRD4 and FAP following JQ1&PFD@CTL treatment (Figure S14, Supporting Information). This suggests that post‐treatment, there is a reduction in BRD4 expression induced by CAFs, likely due to their inhibition. The body weight of the mice remained relatively stable throughout the treatment period, indicating good in vivo safety (Figure 5C). Furthermore, histological evaluations of major organs, including the heart, liver, kidney, and lungs, showed no notable changes in color or texture in each group (Figure S15, Supporting Information). Additionally, blood routine tests, liver function, and kidney function displayed no significant alterations (Figure 5G,H), further supporting the relative in vivo safety of our JQ1&PFD@CTL constructs.

**Figure 6:**
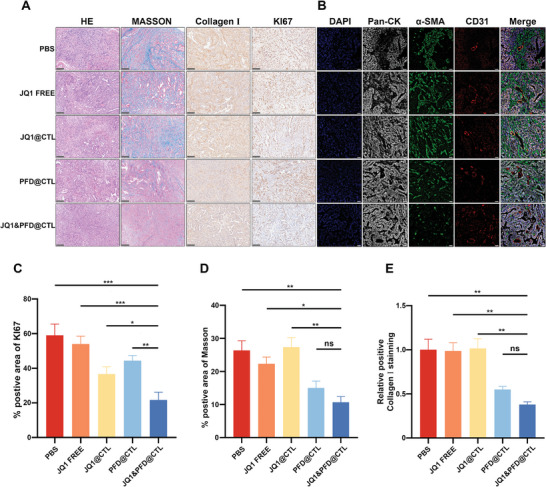
JQ1&PFD@CTL remodeled pancreatic cancer microenvironment in vivo. A) Representative images of tumor HE staining, IHC staining for Ki67 and Collagen I, and Masson's staining were obtained at the end of the experiment, scale bar = 100 µm. B) At the experimental endpoint, tumors were subjected to multi‐color immunofluorescence staining (blue, DAPI; gray, pan‐cytokeratin; green, α‐SMA; red, CD31). Scale bar = 50 µm. C–E) Quantification of collagen content, percentage of Ki67 positivity, and Masson positive area in tumor.

CAFs in the tumor microenvironment regulate the collagen component of the dense extracellular matrix (ECM), establishing a physical barrier that hinders effective drug delivery.^[^ ^33^ ^]^ Hence, the impact of JQ1&PFD@CTL on collagen regulation was investigated. The result of immunohistochemical (IHC) staining showed that the JQ1&PFD@CTL groups effectively suppressed the expression of Ki67 and collagen I, which are markers of tumor proliferation and major constituents of the tumor stroma (**Figure** **6**A). Subsequent histological scoring demonstrated a reduction in Ki67 positivity by 22.33% and 14.67% in the JQ1@CTL and PFD@CTL groups, respectively, compared to the control group. In contrast, JQ1&PFD@CTL treatment resulted in a 37.34% decrease in Ki67 expression (Figure 6C), indicating a potent anti‐proliferative effect. Masson staining and collagen I staining further confirmed that the PFD@CTL and JQ1&PFD@CTL groups effectively downregulated the stromal barrier of the tumor, thereby enhancing drug delivery (Figure 6D,E).

Moreover, the distribution pattern was investigated with immunofluorescence staining, as illustrated in Figure 6B. Significant reduction in α‐SMA expression was observed in the PFD@CTL and JQ1&PFD@CTL groups, indicating the inhibitory effect of PFD on CAFs. Importantly, the tissue samples from the PBS group, free JQ1 group, and JQ1@CTL group exhibited a dense α‐SMA (green fluorescence) positive matrix surrounding the blood vessels (red fluorescence of CD31), which may impede drug penetration into the tumor tissue. In contrast, the stromal wrapping around the blood vessels was weakened in the PFD@CTL and JQ1&PFD@CTL groups, attributed to the inhibitory effect of PFD on the stroma (Figure 6B). This weakened wrapping allowed the perfusion of the drug, improving the efficacy of chemotherapy. Hence, it is believed that the target delivery by JQ1&PFD@CTL inhibits CAFs and stromal barriers, facilitates deeper drug penetration, and enhances JQ1 sensitivity, making it a promising approach for effective cancer treatment.

### 2.6 JQ1&PFD@CTL Reduces Hypoxia and Inhibits Glycolysis for Antitumor Effect

**Figure 7:**
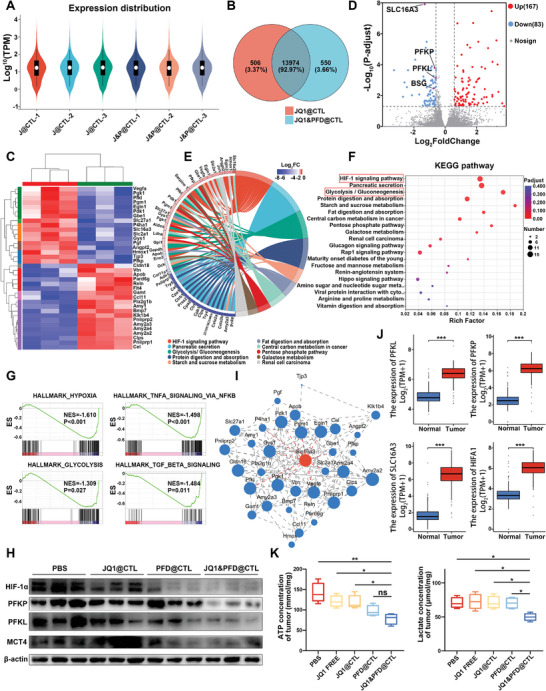
Mechanistic investigation of the antitumor effect of JQ1&PFD@CTL. A) Violin plots of gene expression levels for the gene set. B) Venn diagrams were used to perform sample‐to‐sample correlation analysis for the two groups. The red color represents the JQ1@CTL group, while the blue color represents the JQ1&PFD@CTL group. C) Heatmap was generated to display the representative differentially expressed genes between the JQ1@CTL group and the JQ1&PFD@CTL group. D) RNA sequencing analysis of tumor tissues treated with JQ1@CTL and JQ1&PFD@CTL to identify differentially expressed genes. E,F) KEGG enrichment analysis of signaling pathways. G) Negative enrichment of HYPOXIA, GLYCOLYSIS, TNFA Signaling via NFKB, and TGF Beta Signaling in JQ1&PFD@CTL treated in comparison with the JQ1@CTL via GSEA. We utilized orthology‐mapped hallmark gene sets as gene sets for analysis. H) Western blot analysis was performed to determine the expression of HIF‐1α, PFKP, PFKL, and MCT4 in each group in mouse tumor tissues. I) Gene expression‐related analysis of slc16a3. J) Integrating the TCGA‐PAAD dataset with the normal pancreatic tissue dataset from GTEx, we analyzed the expression levels of PFKP, PFKL, HIF‐1α, and slc16a3 in pancreatic cancer (*n* = 179) and normal pancreatic tissues (*n* = 171). K) Quantitative analysis of ATP levels and lactate levels in each tissue group.

To elucidate the mechanism underlying the anticancer effects of PFD on pancreatic cancer, RNA sequence analysis was conducted on tumor tissues derived from the JQ1&PFD@CTL and JQ1@CTL treatment groups. The violin plot depicting gene expression levels demonstrates relatively consistent gene expression patterns across different samples (**Figure** **7**A). Subsequently, a Venn analysis was performed between the JQ1@CTL and JQ1&PFD@CTL groups, accounting for 92.97% of the total genes with 13974 shared genes (Figure 7B). This finding suggests biological similarities between the JQ1@CTL and JQ1&PFD@CTL groups, indicating that the sample quality is relatively high. The heatmap plot highlights significant differences in gene expression between the JQ1&PFD@CTL group and the JQ1@CTL group (Figure 7C).

Furthermore, KEGG enrichment analysis was employed to investigate the influence of differentially expressed genes, with particular emphasis on the HIF‐1 signaling pathway and glycolysis signaling pathway of the tumor microenvironment (Figure 7E,F). Analysis revealed that nearly all genes enriched in the HIF‐1 signaling pathway exhibited downregulation in the JQ1&PFD@CTL group compared to the JQ1@CTL group, primarily associated with functions related to glycolysis and angiogenesis (Figure 7D). This observation might be attributed to PFD's reduction in extracellular matrix generation, leading to improved oxygen supply and subsequent decrease in HIF‐1 activation.^[^ ^34^ ^]^ GSEA analysis of the RNA‐Seq data demonstrated negative enrichment of HYPOXIA, GLYCOLYSIS, TNFA Signaling via NFKB, and TGF Beta Signaling in the JQ1&PFD@CTL group (Figure 7G). Upon examining all differentially expressed genes, PFKL and PFKP, both encoding Phosphofructokinase, a key enzyme in glycolysis, were among the most significantly downregulated genes in tumor tissues following treatment with JQ1&PFD@CTL. Integration of the TCGA‐PAAD dataset with the GTEx dataset of normal pancreatic tissues enabled an analysis of PFKP and PFKL expression levels in pancreatic cancer. The analysis revealed a significant upregulation of PFKP and PFKL expression in pancreatic cancer compared to normal pancreatic tissues, suggesting a potentially crucial role of PFKP and PFKL in the malignant progression of pancreatic cancer (Figure 7J). Furthermore, a significant reduction in lactate and ATP levels was observed in the JQ1&PFD@CTL group, with a decrease of 29.84% in lactate and 33.58% in ATP compared to JQ1@CTL (Figure 7K). This is attributed to the downregulation of key glycolytic enzymes, indicating inhibition of glycolysis in this group.

**Figure 8:**
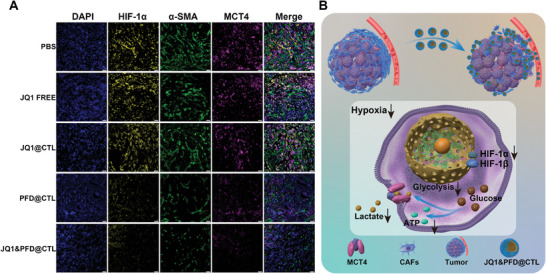
Schematic representation of the anti‐tumor effect of JQ1&PFD@CTL on pancreatic cancer. A) At the experimental endpoint, tumors were subjected to multi‐color immunofluorescence staining (blue, DAPI; yellow, HIF‐1α; green, α‐SMA; violet, MCT4). Scale bar = 50 µm. B) JQ1&PFD@CTL can suppress tumors by inhibiting CAFs and improving the hypoxic environment, thereby inhibiting glycolysis and reducing energy production and lactate metabolism.

Another interesting thing is that a significant downregulation of the slc16a3 gene, which controlled the lactate in the tumor microenvironment, was observed in the JQ1&PFD@CTL group. The protein encoded by slc16a3, known as MCT4, functions as a membrane transporter responsible for the extracellular transport of accumulated lactate from the intracellular compartment, maintaining lactate homeostasis inside and outside the cell.^[^ ^35^ ^]^ Tumor cells upregulate MCT4 expression to enhance lactate transport, adapting to the high‐energy and low‐oxygen environment required for malignant tumor growth.^[^ ^36^ ^]^ The downregulation of slc16a3 may result in intracellular lactate accumulation in tumor cells and a reduction in tumor microenvironment acidity, thereby perturbing the acid‐base balance and metabolism of tumor cells.^[^ ^37^ ^]^ Ultimately, this downregulation inhibits tumor proliferation and growth. We performed immunofluorescence staining on all groups and observed a downregulation of α‐SMA expression in both the PFD@CTL group and JQ1&PFD@CTL group, accompanied by a corresponding downregulation of HIF‐1α and MCT4 expression (**Figure** **8**A). These findings suggest that after matrix inhibition by PFD, oxygen supply is improved, leading to the suppression of the hypoxic inducer HIF‐1α, subsequently inhibiting the glycolytic pathway. Gene expression correlation analysis revealed close associations between slc16a3 and several glycolysis‐related genes (Figure 7I). Analysis of slc16a3 expression in pancreatic cancer demonstrated significant upregulation compared to normal pancreatic tissue (Figure 7J). Additionally, Kaplan–Meier survival analysis revealed that patients with higher levels of slc16a3 expression had a poorer prognosis (Figure S16, Supporting Information).^[^ ^38^ ^]^ Subsequent Western blot experiments confirmed the significant downregulation of MCT4, c‐MYC, PFKP, PFKL, and HIF‐1α expression in the JQ1&PFD@CTL treatment group (Figure 7H and Figure S1D, Supporting Information).

## 3 Conclusion

The tumor microenvironment formed by CAFs represents a critical factor contributing to tumor drug resistance. Our previous research has indicated that CAFs promote proliferation and metastasis in pancreatic tumors, and they can also increase the expression of BRD4 in cancer cells, potentially leading to reduced sensitivity of pancreatic cancer cells to JQ1. Consequently, targeting CAFs may represent a novel therapeutic strategy. In light of this, we have developed co‐modified liposomes (CTL) incorporating CAF cell membrane and TAT peptide targeting CAFs to enhance drug accumulation in pancreatic tumor tissues. Leveraging the homologous targeting capability mediated by the cell membrane, CTL exhibits stronger affinity for tumor tissues, enabling superior drug accumulation at the tumor site. Moreover, the release of PFD within the carrier mitigates the generation of extracellular matrix within the tumor microenvironment, further exposing pancreatic tumors. Simultaneously, the inhibitory effect of PFD on CAFs enhances the sensitivity of cancer cells to JQ1. Transcriptomic analysis of the tissue demonstrates that PFD treatment improves the hypoxic conditions of tumor tissue and downregulates key enzymes and products involved in glycolysis (Figure 8B). These findings suggest that targeting CAFs within the tumor microenvironment can modulate tumor metabolic processes, particularly glucose metabolism and acidity. Consequently, it is believed that the proposed strategy in this study, which integrates antifibrotic drugs and chemotherapeutic agents within a nanocarrier, serves as a promising drug delivery mechanism for augmenting the treatment of drug‐resistant tumors by enhancing drug penetration and improving chemotherapy efficacy.

## 4 Experimental Section

### Cell Culture and Treatment

The MT5 cell line from mouse PDAC was obtained by dissociating tumor cells from KPC transgenic mice, and MT5‐luc was transfected with enhanced firefly luciferase for in situ experiments. NIH/3T3 cells were obtained from the Institute of Biochemistry and Cell Biology, Shanghai Institutes for Biological Sciences, Chinese Academy of Sciences (Shanghai, China). The cells were cultured in a medium containing 10% fetal bovine serum (Invitrogen, Waltham, MA, USA), and identified and tested for mycoplasma contamination using short tandem repeat analysis. MT5, MT5‐luc, and NIH/3T3 cells were maintained in DMEM medium (Invitrogen, Waltham, MA, USA) and cultured at 37 °C in a 5% CO_2_ atmosphere in medium containing 10% fetal bovine serum (Biological Industries, Cromwell, CT, USA), penicillin (100 U mL^−1^), and streptomycin (100 U mL^−1^). The culture medium for MT5‐luc cells contained 0.2% puromycin (2 µg mL^−1^).

### Materials

DSPE‐PEG_2000_ and DSPE‐PEG_2000_‐TAT were synthesized and tested by Ruixi Biologicals Co.Ltd (Xi'an, China), with purity over 95%. DIR and coumarin 6 (C6) were purchased from Aladdin Reagent Database Inc. (Shanghai, China). JQ1 (S71100) was obtained from Selleck (Houston, TX, USA), while PFD (P129335) was purchased from Aladdin Reagent Database Inc. (Shanghai, China). Male mice (C57BL/6J, 18–22 g) were purchased from GemPharmatech Ltd (Jiangsu, China) and acclimated in animal care facilities for 5 days prior to experimentation. All experiments were conducted with approval from the Institutional Animal Care and Use Committee (IACUC) of the Shanghai Institute of Drug Research, Chinese Academy of Sciences. The following antibodies were used for western blotting and immunofluorescence: β‐actin (Sigma‐Aldrich, Cat# A5316), Brd4 (abcam, ab243862), c‐MYC (ProteinTech, 67447‐1‐Ig), α‐SMA (Cell Signaling Technology, Cat# 19 245), Collagen I (abcam, ab260043), HIF‐1α (Cell Signaling Technology, Cat# 48 085), MCT4 (abcam, ab308528), PFKL (Zenbio, R25340), PFKP (Zenbio, R25341), Anti‐rabbit IgG (Cell Signaling Technology, Cat# 7074), and Anti‐mouse IgG (Cell Signaling Technology, Cat# 7076),.

### Preparation and Characterization of Modified Liposomes

Phosphatidylcholine (PC), cholesterol (CHO), and DSPE‐PEG_2000_ were combined in a 100:30:1 ratio and sonicated in 1 mL of ethanol until a clear solution was obtained. For the TL and CTL groups, an additional 1% DSPE‐PEG_2000_‐TAT was added, and the lipid mixture was injected continuously and uniformly into a PBS buffer under magnetic stirring (1000 rpm). The PBS buffer was then stirred for an additional 10 min. In the drug‐laden liposomes, JQ1 and PFD were added or not to 1 mL of ethanol, after which free JQ1 and PFD were separated using an ultrafiltration tube (50 KDa, Millipore).

Cell membranes were isolated from A‐NIH/3T3 cells using the Membrane Protein Extraction Kit (KTP3005, abbkine). The protein content of the purified cell membranes was determined using the BCA protein assay, and the membranes were mixed into the liposome solution at a 1:10 protein‐lipid ratio. Liposomes were extruded through polycarbonate membranes with pore sizes of 400 and 200 nm to prepare CL and CTL. The size distribution and Ζeta‐potential of the liposomes were determined using DLS measurements, and their stability over time was assessed by measuring the mean diameter of the liposomes in PBS (pH 7.4 and 6.2) at different time points over 72 h. The morphology was also imaged using transmission electron microscopy. The encapsulation rate of the measured drug was determined using UV–vis spectrophotometry. Briefly, the ultraviolet absorption peak of JQ1 is at 256 nm, while the ultraviolet absorption peak of PFD is at 315 nm. First, standard concentration curves were obtained using known concentrations of JQ1 and PFD. Subsequently, the tested samples were membrane‐permeabilized with methanol, and the absorbance of JQ1 and PFD was measured at the corresponding absorption peaks. The concentrations of JQ1 and PFD in the samples were calculated using the standard curves. In addition, the in vitro release profiles of JQ1 and PFD were determined by dialysis in PBS at pH 7.4. Briefly, 1 mL of freshly prepared drug‐laden liposomes were sealed in a dialysis bag with a cut‐off molecular weight of 3500 Da, immersed in 50 mL of PBS buffer containing 5.0% Tween, and shaken at 50 rpm at 37 °C. The drug content of the released PBS buffer was determined using UV–vis spectrophotometric analysis, as described above.

### Production of Conditioned Medium

Conditioned medium from CAFs was produced according to previous literature by culturing cells in DMEM medium without FBS under normal conditions until they reached 80% confluency, after which they were cultured for an additional 48 h. For experiments assessing the effects of PFD, cells were cultured in PFD‐containing DMEM medium with 10% FBS for 48 h before being switched to a drug‐free, FBS‐free DMEM medium for an additional 48 h. The collected Conditioned medium was then centrifuged at 2500 × *g* for 15 min, and the supernatant was stored at −80 °C. Prior to use, the Conditioned medium was thawed at 4 °C and supplemented with 10% FBS. Conditioned medium from tumor cells was obtained by centrifuging the tumor supernatant at 2500 × *g* for 15 min and then collecting the supernatant, which was stored at −80 °C.

### Cell Viability Assay

To evaluate cell proliferation, the CCK‐8 assay kit (Tokyo Synergy, Japan) was used. Specifically, cells were seeded at a density of 3 × 10^3^ cells per well in 96‐well plates and treated with drugs or conditioned medium after cell attachment. After 48 h of incubation in a humidified atmosphere containing 5% CO_2_ at 37 °C, cells were further incubated for 1 h at 37 °C with 100 µL of medium supplemented with 10 µL of CCK‐8. The absorbance was measured at 450 nm.

### Colony Formation Assay

To investigate the effects of different treatments on colony formation, MT5 cells were seeded in six‐well plates at a density of 500 cells per well with conditioned media, with or without JQ1. After incubation, cells were fixed with methanol and then washed with phosphate‐buffered saline (PBS). Colonies were stained with 0.1% crystal violet for 20 min, and only colonies containing more than 50 cells were counted as colony‐forming units.

### Migration and Invasion Assays

To determine cell migration and invasion, Transwell and Matrigel invasion assays were performed, respectively. For the transwell migration and invasion assays, MT5 cells treated with different conditioned media were seeded at a density of 1 × 10^4^ cells per well and 2 × 10^4^ cells per well, respectively, in the upper chamber. Seven hundred fifty microliters of DMEM medium containing 20% FBS was added to the lower chamber and the cells from the upper chamber were removed after 24 h. Cells migrating through the membrane to the bottom surface were then fixed in methanol for 15 min, followed by staining with 0.5% crystal violet for 15 min. The number of cells were counted in five different areas using a light microscope at ×200 magnification, and the mean value of migrating or invading cells was expressed as a percentage.

### Wound‐Healing Assay

To perform the scratch assay, MT5 cells were seeded at a concentration of 1 × 10^5^ cells per well in six‐well plates and allowed to reach 90% confluence. A linear scratch wound was then produced by gently scraping the cell monolayer with a 200 µL pipette tip. The original culture medium was then replaced with a different conditioned medium, and the cells were cultured for 24 h. The scratch wounds were visualized and captured using Evos M700 imaging system.

### MCTS Experiment

The surface of a 96‐well plate was coated with 50 µL of 1.5% (w/v) agarose solution (sterilized by wet heat sterilization) to reduce cell adhesion. Next, 100 µL of medium was added to each well. MT5 and NIH/3T3 cells were then collected to generate cell suspensions at a concentration of 1 × 10^5^ cells mL^−1^. MCTS were fabricated from 10 µL cell aggregates, which consisted of the two types of cells in a 1: 1 ratio. After cell sphere formation, DMSO, JQ1, and PFD were added, and the size of the cell spheres was recorded at various time points using Evos M700 imaging system.

### Annexin V‐FITC Apoptosis Assay

To obtain single‐cell suspensions, MCTS (10 spheroids per group) were digested, and the resulting cells were harvested and resuspended in a binding buffer. After incubation with 5 µL fluorescent‐labeled Annexin V and 5 µL PI for 10 min in the dark, the samples were analyzed by flow cytometry, and the data were analyzed using FlowJo v10 software.

### Protein Detection in CL and CTL

To determine the protein profiles, SDS‐PAGE electrophoresis assays were conducted on both CL and CTL samples. In addition, CL and CTL were measured using electrophoresis assays. Liposomes without membrane modifications were used as a control.

### Cellular Uptake

In vitro cellular uptake of liposomes was evaluated in MT5 and NIH/3T3 cells. Coumarin 6 was incorporated during liposome production to enable fluorescent labeling. After the addition of liposomes for 3 or 6 h, cells were washed four times with cold PBS (pH 7.4) and fixed with 4% paraformaldehyde. Cell nuclei were stained with DAPI for 5 min, followed by an additional three washes with PBS. Confocal microscopy was used to capture images for assessing cellular uptake. To further assess the efficiency of cellular uptake, the fluorescence intensity of coumarin 6 in cells was measured using flow cytometry at 3 and 6 h after incubation. Using DiI‐labeled cell membranes to encapsulate DiO‐labeled TL, co‐incubated with cells in culture media at both pH 7.4 and 6.2 for 1 h.

### In Vivo Uptake

A cell suspension was prepared by suspending MT5 cells and NIH/3T3 cells in a 1:1 ratio, resulting in a mixed cell suspension. An equal volume of matrix gel was then added to the mixture. Subsequently, the mixed cell suspension was inoculated into the pancreas of mice at a concentration of 5 × 10^4^ cells per 50 µL. After 2 weeks, a palpable round tumor tissue could be touched under the left rib. Subsequently, liposomes labeled with the near‐infrared fluorescent dye DIR were injected into the mice via the tail vein. The distribution of the liposomes was then imaged 24 h post‐injection using the IndiGO Live Animal Imaging System. The mice were then necropsied and the fluorescence signal was quantified for each organ using the IndiGO imaging system.

### In Vivo In Situ Transplantation Model Pharmacokinetic Study

A cell suspension was prepared by mixing MT5‐luc cells and NIH/3T3 cells in a 1:1 ratio. An equal volume of matrix gel was then added to the cell mixture. Subsequently, the mixed cell suspension was inoculated into the pancreas of mice at a concentration of 5 × 10^4^ cells per 50 µL. Mice were randomly divided into five groups, and tumor growth was monitored by imaging of luciferase. Subsequently, mice were injected with PBS, FREEJQ1, JQ1@CTL, PFD@CTL, and JQ1&PFD@CTL via the tail vein every 2 days for a total of seven injections. The concentration of both JQ1 and PFD was 10 mg kg^−1^. The body weight of the mice was recorded every 3 days. At the end of the treatment period, all mice were euthanized, and tumors and organs, including the heart, liver, kidney, spleen, and lungs, were harvested and analyzed.

### Immunofluorescence Analysis

The NIH/3T3 cells were seeded in 24‐well plates at a density of 3 × 10^4^ cells per well. The cells were then fixed with 4% paraformaldehyde (PFA) for 15 min and permeabilized with 0.5% Triton X‐100 for 15 min. After blocking with 5% bovine serum albumin (BSA) for 1 h at room temperature, the sections were incubated overnight at 4 °C with an antibody against α‐SMA. The images were captured using a confocal microscope.

### Staining of Tissue Sections

Paraffin embedding, sectioning, and staining were performed on the tumor tissue using various techniques, including hematoxylin and eosin (HE), Masson's trichrome, and multi‐fluorescent staining with DAPI, Pan‐Cytokeratin, α‐SMA, and CD31. In addition, Ki67 and Collagen I immunohistochemical analyses were conducted by Servicebio (Wuhan, China).

### Tissue Ribonucleic Acid Sequencing

The tumor tissues were immediately harvested post‐execution and snap‐frozen in liquid nitrogen. Subsequently, the transcriptome was sequenced by Majorbio Co. Ltd. (Shanghai, China).

### ATP and Lactate Measurement

ATP levels and lactate levels were measured using an ATP Determination Kit (Beyotime) and a Micro Lactate Assay Kit (CheKine), respectively, following the manufacturer's instructions.

### Western Blotting Analysis

Cells or tissues were lysed on ice and homogenized with RIPA lysis buffer containing a mixture of protease and phosphatase inhibitors for 30 min. Protein concentrations were determined using the BCA method (Beyotime, China).

### Statistical Analysis

Experimental procedures were repeated at least three times, and the data were presented as mean ± SD. One‐way analysis of variance (ANOVA) was used for multiple comparisons, and Student's *t*‐test was used for comparisons between two groups. Statistical significance was set at *p < 0.05, **p < 0.01, ***p < 0.001.

### Ethical Approval

All the animal experiments complied with the Institutional Animal Care and Use Committee of Nanjing Drum Tower Hospital (20201105).

## Conflict of Interest

The authors declare no conflict of interest.

## Author Contributions

Yin Z., R.Y., and C.Z. contributed equally to this work. Yin Z. performed conceptualization, methodology, validation, formal analysis, investigation, data curation, validation, software, wrote the original draft, and reviewed and edited the final manuscript. R.Y. performed validation, formal analysis, investigation, data curation, validation, software, and wrote the original draft. C.Z. performed validation, formal analysis, investigation, data curation, visualization, and wrote the original draft. J.L. and H.S. performed supervision, conceptualization, investigation, and reviewed and edited the final manuscript. Yixuan Z. performed supervision, conceptualization, investigation, and reviewed and edited the final manuscript. J.Z. and H.W. performed investigation, validation, and visualization. S.X. was associated with formal analysis, validation, methodology, and software. Z.Z. was associated with formal analysis, validation, and methodology. L.W. and X.Z. contributed to: resources and funding acquisition. Yun Z. performed conceptualization, methodology, resources, supervision, project administration, funding acquisition, and reviewed and edited the final manuscript. S.Z. and Y.L. performed conceptualization, methodology, project administration, funding acquisition, acquired resources, and reviewed and edited the final manuscript.

## Supporting information

Supporting Information

## Data Availability

The data that support the findings of this study are available from the corresponding author upon reasonable request.

## References

[1] a) B. Ren , M. Cui , G. Yang , H. Wang , M. Feng , L. You , Y. Zhao , Mol Cancer 2018, 17, 108; 30060755 10.1186/s12943-018-0858-1 PMC6065152

[2] R. Siegel , J. Ma , Z. Zou , A. Jemal , Ca‐Cancer J. Clin. 2014, 64, 9. 24399786 10.3322/caac.21208

[3] a) B. Donati , E. Lorenzini , A. Ciarrocchi , Mol Cancer 2018, 17, 164; 30466442 10.1186/s12943-018-0915-9 PMC6251205

[4] M. Pervaiz , P. Mishra , S. Günther , Chem. Rec. 2018, 18, 1808. 30289209 10.1002/tcr.201800074

[5] X. Jin , Y. Yan , D. Wang , D. Ding , T. Ma , Z. Ye , R. Jimenez , L. Wang , H. Wu , H. Huang , Mol. Cell 2018, 71, 592. 30057199 10.1016/j.molcel.2018.06.036 PMC6086352

[6] W. Wang , Y.‐A. Tang , Q. Xiao , W. C. Lee , B. Cheng , Z. Niu , G. Oguz , M. Feng , P. L. Lee , B. Li , Z.i‐H. Yang , Y.‐F. Chen , P. Lan , X.‐J. Wu , Q. Yu , Nat. Commun. 2021, 12, 4441. 34290255 10.1038/s41467-021-24687-4 PMC8295257

[7] a) S. Raghavan , P. S. Winter , A. W. Navia , H. L. Williams , A. Denadel , K. E. Lowder , J. Galvez‐Reyes , R. L. Kalekar , N. Mulugeta , K. S. Kapner , M. S. Raghavan , A. A. Borah , N. Liu , S. A. Väyrynen , A. D. Costa , R. W. S. Ng , J. Wang , E. K. Hill , D. Y. Ragon , L. K. Brais , A. M. Jaeger , L. F. Spurr , Y. Y. Li , A. D. Cherniack , M. A. Booker , E. F. Cohen , M. Y. Tolstorukov , I. Wakiro , A. Rotem , B. E. Johnson , et al., Cell 2021, 184, 6119; 34890551 10.1016/j.cell.2021.11.017 PMC8822455

[8] J. P. Neoptolemos , J. Kleeff , P. Michl , E. Costello , W. Greenhalf , D. H. Palmer , Nat Rev Gastroenterol Hepatol 2018, 15, 333. 29717230 10.1038/s41575-018-0005-x

[9] P. P. Provenzano , C. Cuevas , A. E. Chang , V. K. Goel , D. D. Von Hoff , S. R. Hingorani , Cancer Cell 2012, 21, 418. 22439937 10.1016/j.ccr.2012.01.007 PMC3371414

[10] E. Sahai , I. Astsaturov , E. Cukierman , D. G. Denardo , M. Egeblad , R. M. Evans , D. Fearon , F. R. Greten , S. R. Hingorani , T. Hunter , R. O. Hynes , R. K. Jain , T. Janowitz , C. Jorgensen , A. C. Kimmelman , M. G. Kolonin , R. G. Maki , R. S. Powers , E. Puré , D. C. Ramirez , R. Scherz‐Shouval , M. H. Sherman , S. Stewart , T. D. Tlsty , D. A. Tuveson , F. M. Watt , V. Weaver , A. T. Weeraratna , Z. Werb , Nat Rev Cancer 2020, 20, 174. 31980749 10.1038/s41568-019-0238-1 PMC7046529

[11] J. Behr , A. Prasse , M. Kreuter , J. Johow , K. F. Rabe , F. Bonella , R. Bonnet , C. Grohe , M. Held , H. Wilkens , P. Hammerl , D. Koschel , S. Blaas , H. Wirtz , J. H. Ficker , W. Neumeister , N. Schönfeld , M. Claussen , N. Kneidinger , M. Frankenberger , S. Hummler , N. Kahn , S. Tello , J. Freise , T. Welte , P. Neuser , A. Günther , J. Behr , M. Kreuter , J. Johow , et al., Lancet Respir Med 2021, 9, 476.33798455

[12] a) J. Zhao , Y. Zhu , Z. Li , J. Liang , Y. Zhang , S. Zhou , Y. Zhang , Z. Fan , Y. Shen , Y. Liu , F. Zhang , S. Shen , G. Xu , L. Wang , Y. Lv , S. Zhang , X. Zou , Biomater. Sci. 2022, 10, 6614; 36260512 10.1039/d2bm00770c

[13] B. Ferrara , C. Pignatelli , M. Cossutta , A. Citro , J. Courty , L. Piemonti , Cancers 2021, 13. 34503252 10.3390/cancers13174442 PMC8430646

[14] J. Tao , G. Yang , W. Zhou , J. Qiu , G. Chen , W. Luo , F. Zhao , L. You , L. Zheng , T. Zhang , Y. Zhao , J. Hematol. Oncol. 2021, 14, 14. 33436044 10.1186/s13045-020-01030-w PMC7805044

[15] a) T. Ji , S. Li , Y. Zhang , J. Lang , Y. Ding , X. Zhao , R. Zhao , Y. Li , J. Shi , J. Hao , Y. Zhao , G. Nie , ACS Appl. Mater. Interfaces 2016, 8, 3438; 26759926 10.1021/acsami.5b11619

[16] a) X. Duan , C. Chan , W. Lin , Angew. Chem., Int. Ed. 2019, 58, 670; 10.1002/anie.201804882 PMC7837455 30016571

[17] a) A. N. Hosein , R. A. Brekken , A. Maitra , Nat Rev Gastroenterol Hepatol 2020, 17, 487; 32393771 10.1038/s41575-020-0300-1 PMC8284850

[18] G. Biffi , D. A. Tuveson , Physiol Rev 2021, 101, 147. 32466724 10.1152/physrev.00048.2019 PMC7864232

[19] R. Kalluri , Nat Rev Cancer 2016, 16, 582. 27550820 10.1038/nrc.2016.73

[20] a) J. Li , X.u Zhen , Y. Lyu , Y. Jiang , J. Huang , K. Pu , ACS Nano 2018, 12, 8520; 30071159 10.1021/acsnano.8b04066

[21] R. H. Fang , W. Gao , L. Zhang , Nat Rev Clin Oncol 2023, 20, 33. 36307534 10.1038/s41571-022-00699-x

[22] H. Brooks , B. Lebleu , E. Vives , Adv. Drug Delivery Rev. 2005, 57, 559. 10.1016/j.addr.2004.12.001 15722164

[23] W. Yang , J. Soares , P. Greninger , E. J. Edelman , H. Lightfoot , S. Forbes , N. Bindal , D. Beare , J. A. Smith , I. R. Thompson , S. Ramaswamy , P. A. Futreal , D. A. Haber , M. R. Stratton , C. Benes , U. McDermott , M. J. Garnett , Nucleic Acids Res. 2013, 41, D955. 23180760 10.1093/nar/gks1111 PMC3531057

[24] J. Peng , B.‐F.a Sun , C.‐Y. Chen , J.‐Y.i Zhou , Y.u‐S. Chen , H. Chen , L. Liu , D. Huang , J. Jiang , G.‐S. Cui , Y. Yang , W. Wang , D. Guo , M. Dai , J. Guo , T. Zhang , Q. Liao , Y.i Liu , Y.‐L. Zhao , D.a‐L.i Han , Y. Zhao , Y.‐G. Yang , W. Wu , Cell Res. 2019, 29, 725. 31273297 10.1038/s41422-019-0195-y PMC6796938

[25] H. Janouskova , G. El Tekle , E. Bellini , N. D. Udeshi , A. Rinaldi , A. Ulbricht , T. Bernasocchi , G. Civenni , M. Losa , T. Svinkina , C. M. Bielski , G. V. Kryukov , L. Cascione , S. Napoli , R. I. Enchev , D. G. Mutch , M. E. Carney , A. Berchuck , B. J. N. Winterhoff , R. R. Broaddus , P. Schraml , H. Moch , F. Bertoni , C. V. Catapano , M. Peter , S. A. Carr , L. A. Garraway , P. J. Wild , J.‐P. P. Theurillat , Nat. Med. 2017, 23, 1046. 28805821 10.1038/nm.4372 PMC5592092

[26] Y. Zhu , L. Wen , S. Shao , Y. Tan , T. Meng , X. Yang , Y. Liu , X. Liu , H. Yuan , F. Hu , Biomaterials 2018, 161, 33. 29421561 10.1016/j.biomaterials.2018.01.023

[27] M. Tarannum , K. Holtzman , D. Dréau , P. Mukherjee , J. L. Vivero‐Escoto , J. Controlled Release 2022, 347, 425. 10.1016/j.jconrel.2022.05.019 35569588

[28] M. H. Sherman , G. L. Beatty , Annu Rev Pathol 2023, 18, 123. 36130070 10.1146/annurev-pathmechdis-031621-024600 PMC9877114

[29] Y. Ma , K. Poole , J. Goyette , K. Gaus , Front Immunol 2017, 8, 1513. 29170669 10.3389/fimmu.2017.01513 PMC5684113

[30] a) D. Öhlund , A. Handly‐Santana , G. Biffi , E. Elyada , A. S. Almeida , M. Ponz‐Sarvise , V. Corbo , T. E. Oni , S. A. Hearn , E. J. Lee , I. I.n C. Chio , C.‐I.l Hwang , H. Tiriac , L. A. Baker , D. D. Engle , C. Feig , A. Kultti , M. Egeblad , D. T. Fearon , J. M. Crawford , H. Clevers , Y. Park , D. A. Tuveson , J. Exp. Med. 2017, 214, 579; 28232471 10.1084/jem.20162024 PMC5339682

[31] X. Mao , J. Xu , W. Wang , C. Liang , J. Hua , J. Liu , B.o Zhang , Q. Meng , X. Yu , S.i Shi , Mol Cancer 2021, 20, 131. 34635121 10.1186/s12943-021-01428-1 PMC8504100

[32] a) H. Fatma , S. K. Maurya , H. R. Siddique , Semin. Cancer Biol. 2022, 83, 166; 33220458 10.1016/j.semcancer.2020.11.008

[33] F. Guo , Y. Jiao , Y. Du , S. Luo , W. Hong , Q. Fu , A. Li , G. Wang , G. Yang , Int. J. Biol. Macromol. 2022, 220, 1133. 35988724 10.1016/j.ijbiomac.2022.08.123

[34] a) S.‐B.o Wang , Z.‐X. Chen , F. Gao , C. Zhang , M.‐Z. Zou , J.‐J. Ye , X. Zeng , X.‐Z. Zhang , Biomaterials 2020, 234, 119772; 31945618 10.1016/j.biomaterials.2020.119772

[35] Y. Contreras‐Baeza , P. Y. Sandoval , R. Alarcón , A. Galaz , F. Cortés‐Molina , K. Alegría , F. Baeza‐Lehnert , R. Arce‐Molina , A. Guequén , C. A. Flores , A. San Martín , L. F. Barros , J Biol Chem 2019, 294, 20135. 31719150 10.1074/jbc.RA119.009093 PMC6937558

[36] G. Baek , Y. F. Tse , Z. Hu , D. Cox , N. Buboltz , P. Mccue , C. J. Yeo , M. A. White , R. J. Deberardinis , E. S. Knudsen , A. K. Witkiewicz , Cell Rep. 2014, 9, 2233. 25497091 10.1016/j.celrep.2014.11.025

[37] X. Li , Y. Yang , B. Zhang , X. Lin , X. Fu , Y.i An , Y. Zou , J.‐X. Wang , Z. Wang , T. Yu , Signal Transduction Targeted Ther. 2022, 7, 305. 10.1038/s41392-022-01151-3 PMC9434547 36050306

[38] Z. Tang , B. Kang , C. Li , T. Chen , Z. Zhang , Nucleic Acids Res. 2019, 47, W556. 31114875 10.1093/nar/gkz430 PMC6602440

