# Supplementary material for: CAFs Homologous Biomimetic Liposome Bearing BET Inhibitor and Pirfenidone Synergistically Promoting Antitumor Efficacy in Pancreatic Ductal Adenocarcinoma
Source: Adv Sci (Weinh). 2023 Nov 15;11(1):2305279. doi: 10.1002/advs.202305279 (PMC10767438; doi:10.1002/advs.202305279)
Supplement: Supplementary file 1 — Supporting Information [file ADVS-11-2305279-s001.pdf]

ADVANCED SCIENCE
Open Access

Supporting Information

for *Adv. Sci.*, DOI 10.1002/advs.202305279

CAFs Homologous Biomimetic Liposome Bearing BET Inhibitor and Pirfenidone Synergistically Promoting Antitumor Efficacy in Pancreatic Ductal Adenocarcinoma

Yin Zhang, Ranran Yu, Cheng Zhao, Jiawei Liang, Yixuan Zhang, Haochen Su, Jing Zhao, Hao Wu, Shijin Xu, Ziying Zhang, Lei Wang, Xiaoping Zou, Yun Zhu, Shu Zhang* and Ying Lv**

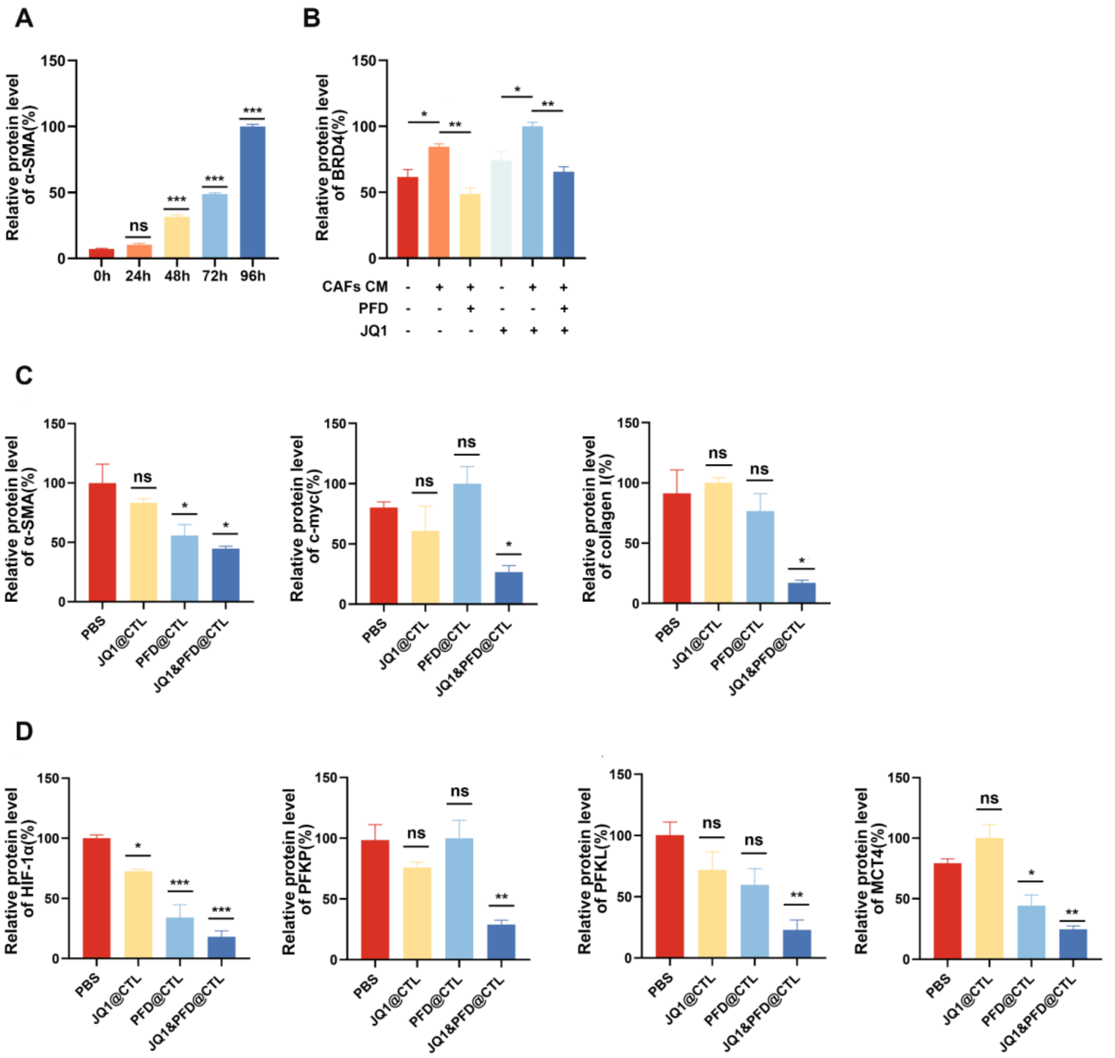

Figure S1. (A) The relative quantification of α-SMA expression in NIH/3T3 cells at different time points after treatment with conditioned medium collected from MT5 cells in Figure 2A was performed. **(B)** The relative quantification of BRD4 expression in MT5 cells treated with JQ1 and CAFs or PFD-treated CAFs conditioned medium in Figure 2G was performed. **(C)** Relative quantification of α-SMA, Collagen I, and c-myc expression in each group in mouse tumor tissues in Figure 5F was performed. **(D)** Relative quantification of HIF-1α, PFKP, PFKL and MCT4 expression in each group in mouse tumor tissues in Figure 7H was performed. The data are presented as mean ± SD from three independent experiments. $^*P < 0.05$, $^{**}P < 0.01$, $^{***}P < 0.001$.

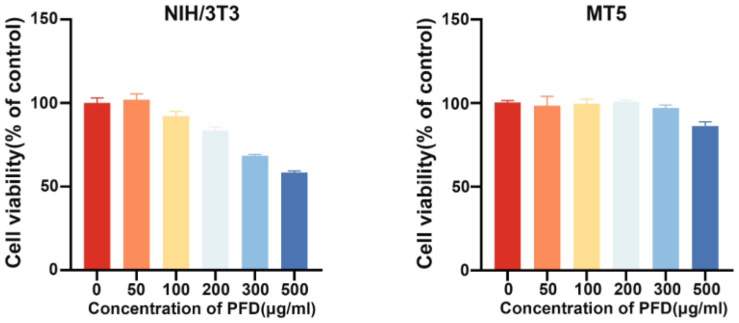

Figure S2. CCK8 test for the evaluation of proliferation of MT5 and NIH/3T3 treated by PFD at different concentration for 48h. The data are presented as mean ± SD from three independent experiments. *P < 0.05, **P < 0.01, ***P < 0.001.

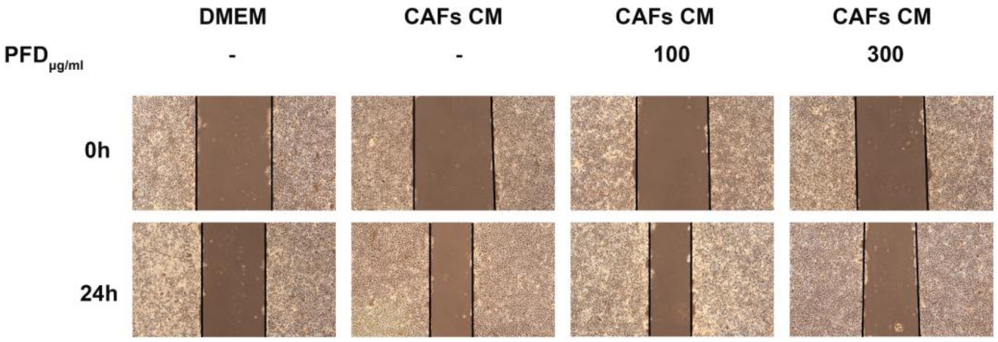

Figure S3. Wound healing assay of MT5 cells treated with conditioned medium from CAFs or PFD-treated CAFs.

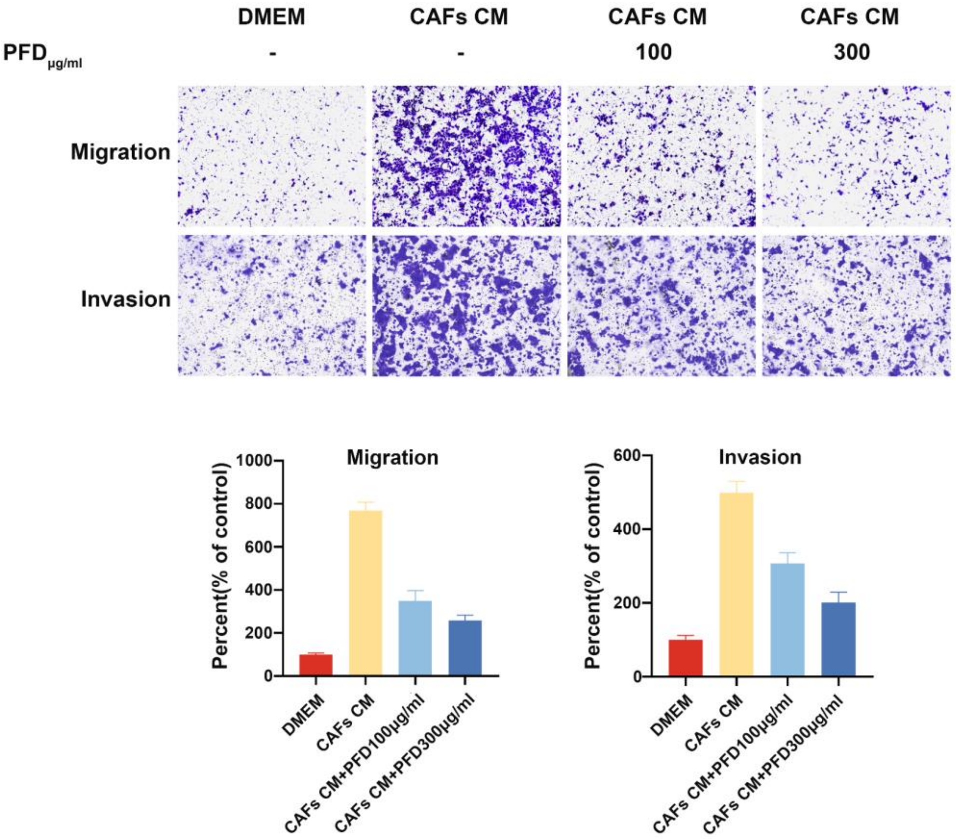

Figure S4. Migration and invasion of MT5 cells treated with conditioned media of CAFs or PFD-treated CAFs and quantitative analysis. The data are presented as mean ± SD from three independent experiments. $^{*}P < 0.05$, $^{**}P < 0.01$, $^{***}P < 0.001$.

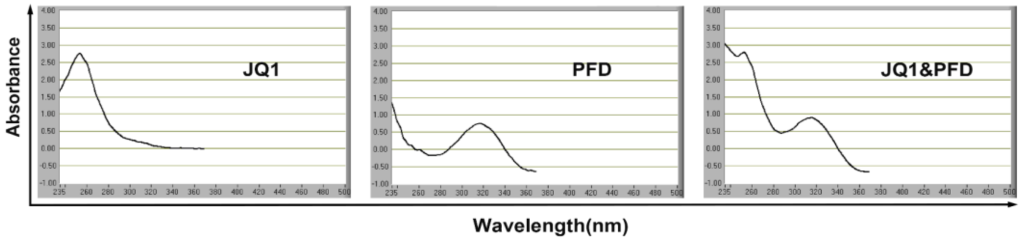

Figure S5. Drug absorption peaks of JQ1 and PFD measured by UV-vis spectrophotometer.

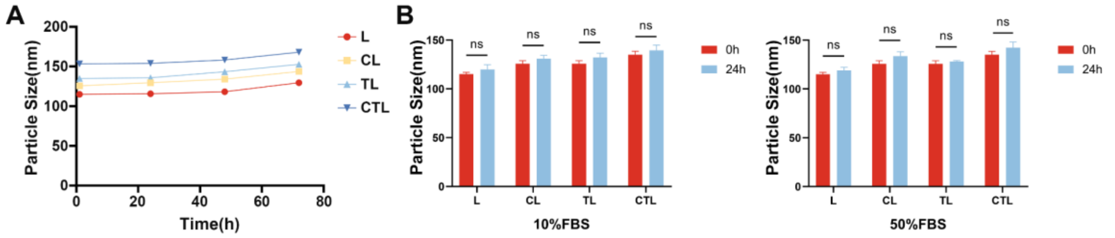

Figure S6. (A) Changes in the average diameter of L, CL, TL, and CTL in PBS with a pH value of 7.4 at various time points within 72 hours. **(B)** Changes in the average diameter of L, CL, TL, and CTL within 24 hours under conditions of 10% FBS and 50% FBS. The data are presented as mean ± SD from three independent experiments. *P < 0.05, **P < 0.01, ***P < 0.001.

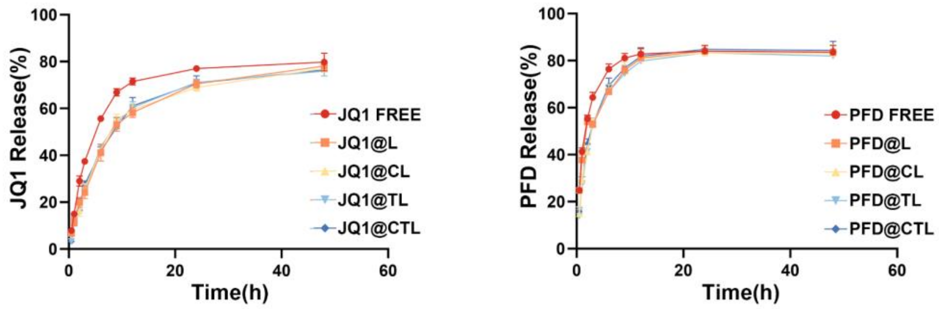

Figure S7. In vitro cumulative drug release of the liposome in PBS at PH 6.2. The data are presented as mean ± SD from three independent experiments. *P < 0.05, **P < 0.01, ***P < 0.001.

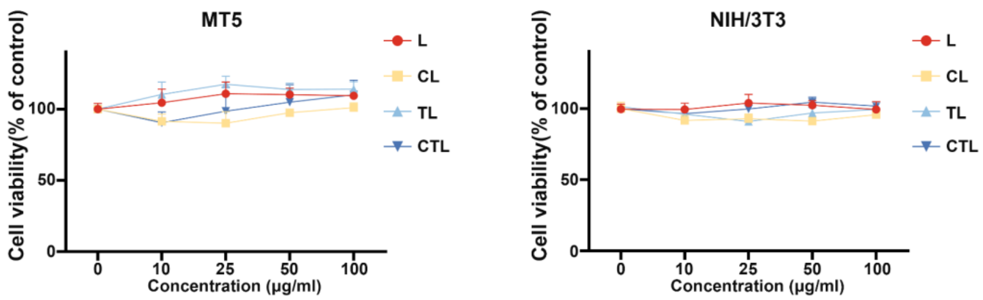

Figure S8. The cytotoxicity of blank liposome in MT5 and NIH/3T3 cells. The data are presented as mean ± SD from three independent experiments. *P < 0.05, **P < 0.01, ***P < 0.001.

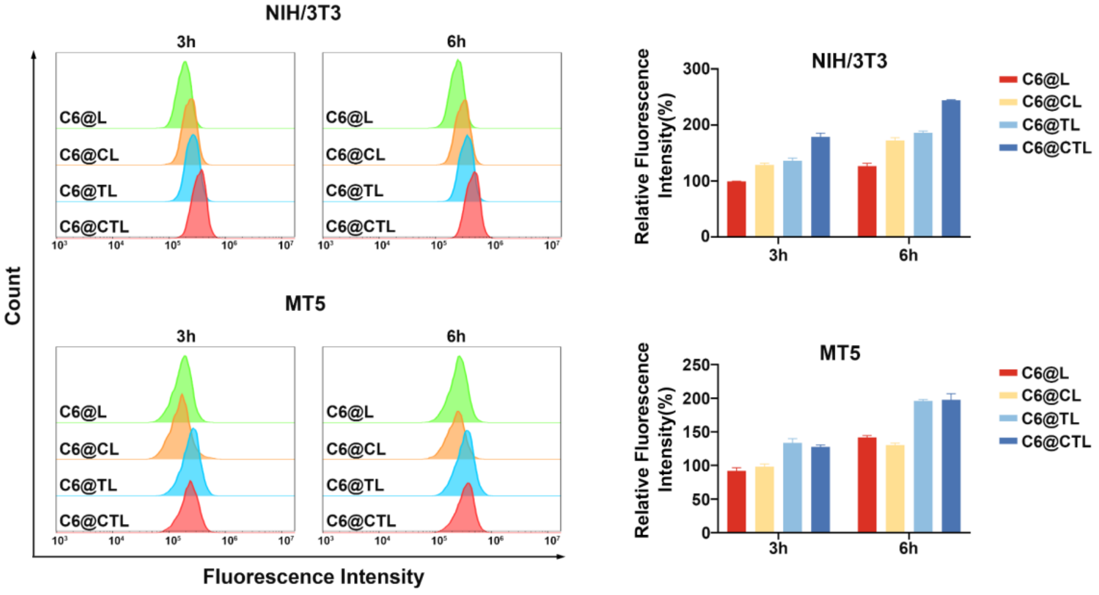

Figure S9. Flow cytometry showed intracellular uptake of C6@L, C6@CL, C6@TL, C6@CTL in MT5 and NIH/3T3 cells. The data are presented as mean ± SD from three independent experiments. *P < 0.05, **P < 0.01, ***P < 0.001.

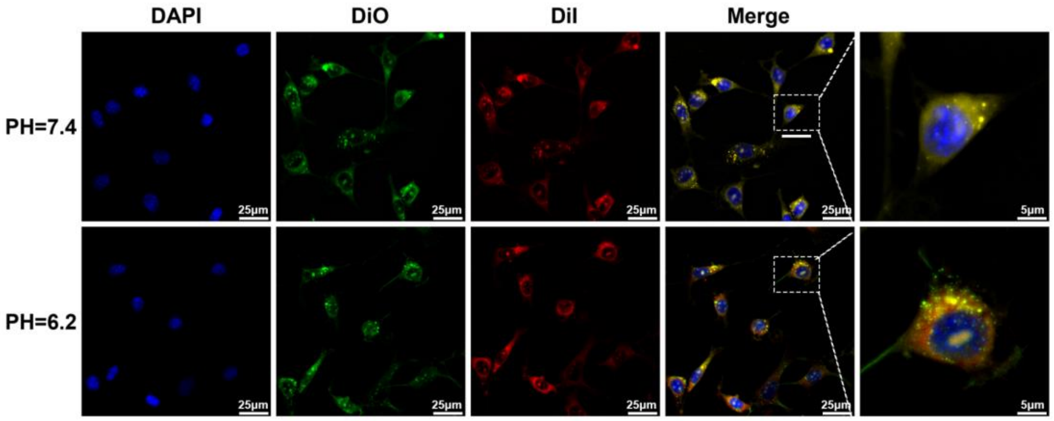

Figure S10. Confocal microscopy images showing the entry of fluorescently labeled CTL into NIH/3T3 cells one hour after incubation in cell culture media with different pH levels. The green fluorescence signal represents TL, while the red fluorescence signal represents the cell membrane.

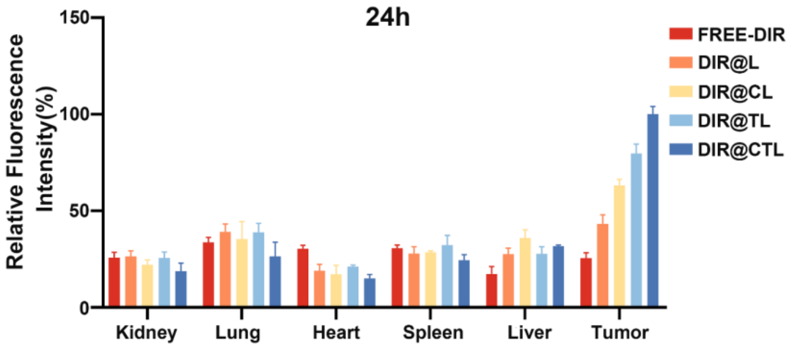

Figure S11. Quantification of fluorescence intensity in different organs of FREE-DIR, DIR@L, DIR@CL, DIR@TL, and DIR@CTL groups 24 hours after injection. The data are presented as mean ± SD from three independent experiments. *P < 0.05, **P < 0.01, ***P < 0.001.

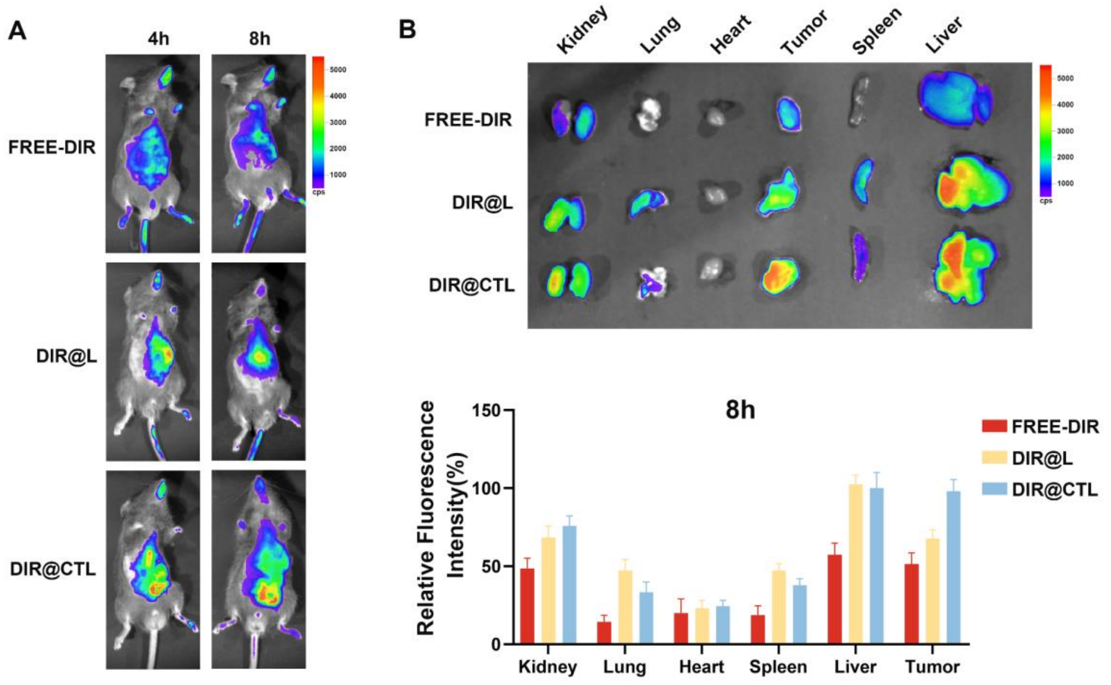

Figure S12. (A) Fluorescence intensity in vivo detected using the IndiGO imaging system at 4 and 8 hours after injection of FREE-DIR, DIR@L and DIR@CTL. **(B)** Fluorescence intensity and quantification in various organs of FREE-DIR, DIR@L and DIR@CTL groups at 8 hours post-injection. The data are presented as mean ± SD from

three independent experiments. *P < 0.05, **P < 0.01, ***P < 0.001.

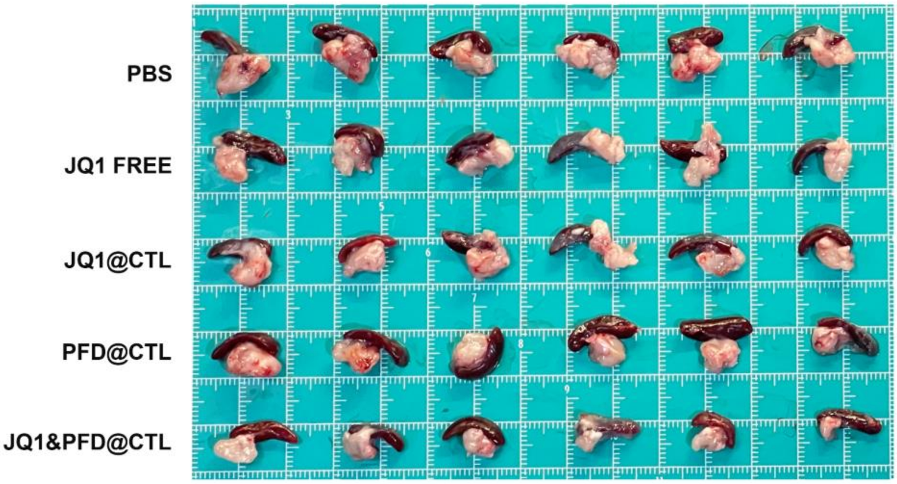

Figure S13. Tumor and spleen dissected after humanitarian execution.

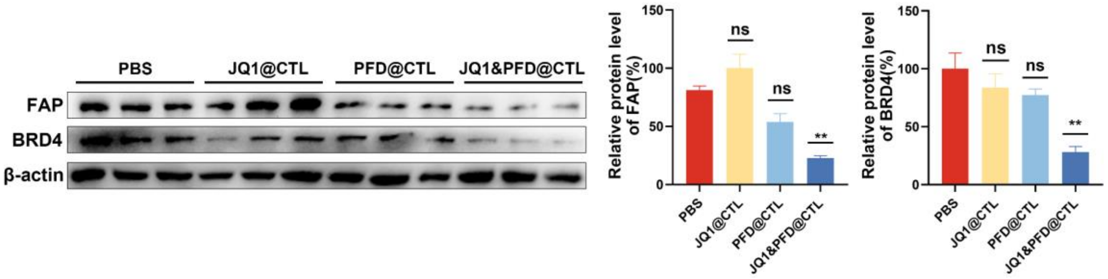

Figure S14. Western blot analysis was conducted to evaluate the expression of FAP and BRD4 in each group in mouse tumor tissues. The data are presented as mean ± SD from three independent experiments. *P < 0.05, **P < 0.01, ***P < 0.001.

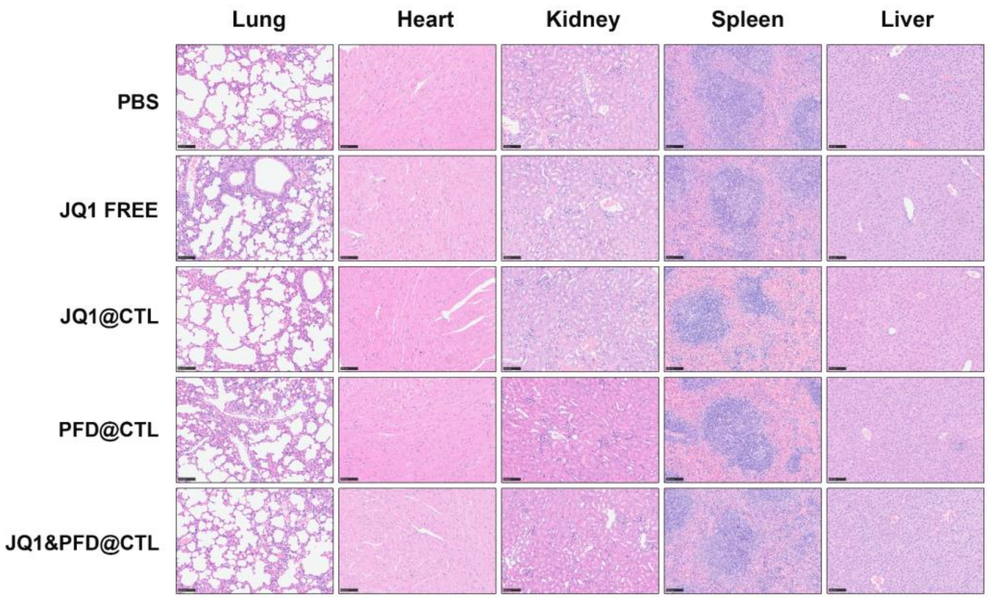

Figure S15. HE staining of the heart, liver, kidney, spleen and lung tissues in mice, scale bar = 100μm.

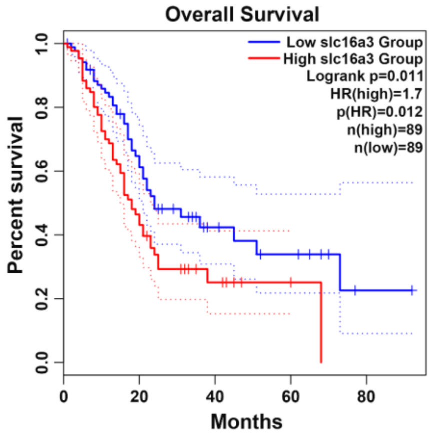

Figure S16. Kaplan-Meier survival analysis showing the relationship between slc16a3 expression levels and prognosis in pancreatic cancer.
